# Medicinal plants of the upper Aswa River catchment of northern Uganda - a cultural crossroads

**DOI:** 10.1186/s13002-023-00620-5

**Published:** 2023-10-27

**Authors:** Eliot T. Masters

**Affiliations:** Nelson Marlborough Institute of Technology (Te Pūkenga), Nelson, New Zealand

**Keywords:** Ethnobotany, Ethnomedicine, Ethnopharmacology, Phytomedicines, Plant-based medicine, Cultural comparison, Northern Uganda

## Abstract

**Background:**

This paper presents a comparative inventory of medicinal plant taxa and their uses by smallholder farming communities of four cultures in the Aswa River catchment of northern Uganda, situated in the eastern Sudanian savanna parkland ecotype of sub-Saharan Africa. The purpose of the study was to document the ethnobotanical use of medicinal plants by the Lango, Acholi, Teso (Atesot) and Ethur (jo Abwor), in an historical moment before civil conflict and mass displacement of the respondent communities disrupted the inter-generational transmission of traditional technical knowledge within the study area.

**Methods:**

Following community consultations in four districts of northern Uganda during 1999–2000, interviews were conducted with holders of specialist knowledge on plants used as medicine on basis of a plant specimen allocated a voucher number and identified by the national herbarium. Use reports reflecting specific medicinal applications were compiled in aggregate to obtain a Relative Importance Index ranking. The commonality of medicinal taxa cited between each cultural interface was assessed by the Jaccard Index of Similarity, and the similarity of specific medicinal usage by taxon using Rahman’s Similarity Index.

**Results:**

The data collected from 112 respondents comprise 280 medicinal use reports describing 263 applications for 62 medical conditions, citing 108 taxa from 44 botanical families of which Fabaceae comprised 20% of all use reports. No earlier mention could be found to corroborate 72 use reports (27% of the total), representing medicinal indications as yet undocumented, and potentially worthy of investigation. The RI values ranged between 15 and 94%, with 13 taxa having RI values above 50%. The JI ratios indicate the highest degree of similarity in the plant taxa used as medicine (21%) between the Lango and Teso cultures who share a common origin; however, Rahman’s Similarity Index indicates the highest similarity of specific medicinal usage by taxon between the Lango and Acholi, who share a common language group through cultural assimilation over time.

**Conclusions:**

As a comparative study, the results imply that cultural exchange and assimilation may be a greater driver of inter-cultural similarity of ethnopharmacological use of a given taxon, as compared to shared historical origins.

**Supplementary Information:**

The online version contains supplementary material available at 10.1186/s13002-023-00620-5.

## Background

Sustained by plant foods from our earliest origins, human use of plant medicines predates our earliest ancestry—and indeed our species—as a deep evolutionary behavior [1]. The plant kingdom has long provided panacea for human discomforts and diseases, as evidenced by archeological traces extending back as far as 1.2 million years ago [1]—and, much later, in the earliest of written records [2]. Of an estimated 390,900 plant species of record, over 28,000 have a recorded medicinal use [3]. Plant-derived molecules provide the basis for development of modern synthetic pharmaceuticals, and medicines derived directly from plants still constitute a quarter of the modern pharmacopeia [4].

Plant-based medicines are highly valued globally—more particularly for rural communities in sub-Saharan Africa, where conventional medical services are largely inaccessible and unaffordable—including rural northern Uganda. The World Health Organization (WHO) estimates that up to 80% of the global population relies on plant compounds in primary healthcare, while traditional medicines are becoming ever more popular as complementary therapies [5]. Based on a national-level survey, it has been estimated that 60% of Ugandans used traditional medicine, of which an estimated 90% was plant-based, noting that plant-based medicines are both more affordable than conventional therapies, and also more readily accessible—with a 1:700 ratio of traditional healers to population, versus a 1:25,000 ratio of medical doctors [6]. 

However, even as burgeoning global demand for medicinal plants in complementary treatments has come to threaten the survival of certain species [7], traditional knowledge of medicinal plant use is steadily receding from living memory, with inter-generational knowledge transfer declining further each year [8], in an accelerating process which has been termed cultural erosion [9]. 

In northern Uganda, this ongoing process was drastically accelerated by civil conflict in the early years of the current century, resulting in mass displacement of the civilian population into military camps just two years following data collection, resulting in severe disruption to traditional agriculture and transmission of indigenous knowledge on the use of plant biodiversity by rural communities [10]. The return of peace to the region (and the displaced to their homesteads) in 2007 brought other, demographic changes and socioeconomic challenges. Although Uganda is categorically an agricultural country, with 39% of households situated within the subsistence economy, its young and growing population—54% below 18 years—is online, aspirational and increasingly disinclined see their future in traditional agriculture [11]. In this socio-cultural context, the transmission of traditional knowledge can be considered as highly threatened.

Conceived as an inventory of taxa and their pharmacological use by culture, the operational hypothesis behind the study was that, given their similar geographies along a gradient of reduced rainfall from southwest to northeast, patterns of plant use within and between the four cultures surveyed would be similar, but also characteristically different—and that the relative degrees of relatedness of medicinal plant use between the four communities might be expected to reflect ethnic as well as cultural dimensions of their shared historical proximity. 

The aim of the broader study, conducted under the auspices of an integrated conservation and development project aimed at documenting and reinforcing sustainable use of the tree and other woodland plant species, was to compile a full inventory of plant species traditionally utilized by rural communities, drawing upon the expertise and interest of key informants, and leveraging tacit knowledge from those motivated to share it in order to codify plant utilization and its management. This was achieved through a series of multi-dimensional (cultural, ethnographic, socioeconomic, ecological and botanical) studies of plant diversity, its use and management by smallholder farming households across the project area, of which this paper presents the results relevant to the use of medicinal plants.

## Methods

### The study area, landscapes and communities

The study area lies between 1° and 3° North and from 33° to 34° degrees East, comprising the southern catchment of the Aswa^1^ River, a major tributary of the Nile which rises from the Labwor Hills and other inselbergs punctuating wooded parkland on sandy loams over a lateritic ironstone layer [12]. At elevations ranging between 1025 and 1300 m, with unimodal rainfall between 750 and 1500 mm from April to October, and temperatures mostly ranging between 16° and 32°C [13], the study area corresponds to the Sudanian ecotype, which extends from eastern Senegal to the Gambella region of Ethiopia, and to Lake Kyoga at the geographic center of Uganda. 

The plant biodiversity of Uganda as a whole, and of the study area in particular, has been documented historically—classified and mapped according to vegetation type at a scale of 1:500,000 [14], the central northern Uganda region characterized by a wooded savanna dominated by *Vitellaria paradoxa* C.F.Gaertn. subsp. *nilotica* (Kotschy) A.N.Henry, Chithra & N.C.Nair (formerly *Butyrospermum paradoxum* (C.F.Gaertn.) Hepper subsp. *niloticum* (Kotschy) Hepper) in association with other woody and herbaceous species (notably the savanna grass *Hyparrhenia rufa* Stapf.) The author participated in ground truthing of the 1:50,000 satellite data for revalidation of this floristic baseline in 1992–93 [15], which facilitated targeting of the study area for applied research on basis of its relative ecological integrity as reflected in the density of indigenous woodland.

The woodland species documented in the study area (and reflected in the use reports presented here) are found in similar association across this narrow band of Sudanian savanna [16], of which the shea butter tree *Vitellaria paradoxa* subsp. *nilotica* can be considered a cultural keystone species as defined by Garibaldi and Turner [17]. *V. paradoxa* is an indicator species of the parkland agroforestry system [18], characterized by the diversity of tree species conserved on farm when fallow woodland is cleared for cultivation [19], resulting a mosaic of cultivation embedded within a wooded landscape. According to the comprehensive review of earlier studies, the ethnopharmacology of the Sudanian ecotype and its resident cultures has been found to be distinctly under-represented in the ethnomedicinal literature as compared to the more humid forest biomes.

The respondent communities of the study area are distributed between the trading centers among rural hamlets, where households and communities remain profoundly connected to the plant biodiversity and its environmental services on which their livelihoods depend. As of 2014, the proportion of households engaged in subsistence farming as a main source of livelihood comprised 90.6% of the population of Otuke District [20], 91.1% of Agago [21], 85% of Amuria [22], and 86.7% of Abim [23]. Poverty is notably and pervasively higher in northern Uganda, literacy and access to sanitation notably lower than in other regions of the country [24].

The people of the study area—the Lango, Acholi, Teso (Atesot) and Ethur (jo Abwor)—are situated across four historical regions, comprising four distinct ethnicities, cultures and dialects across two major language groups. Each community recalls an early history of migration from a northern homeland to reside around an historic cultural boundary, or interface, represented by the massif of Mount Otuke—the remnant of an ancient granitic inselberg ridge, oriented northwest-southeast along the boundary between the present-day districts of Otuke and Abim (formerly Labwor, a name more reflective of its eponymous hills). 

In its location and shared history, Mount Otuke represents a cultural frontier between the Lwo-origin Acholi and Ethur to the north, and the ‘Paranilotic’ Ateker-origin Teso and Lango to the south and east. Each of the four cultures recalls Mount Otuke as an ancestral hub or heartland around which they migrated in early times (a period known as ***aconya*** in the Lwo languages, ***asonya*** in Teso), particularly during the latter half of the 1700s [25]. At an elevation of 1400 m, the saddle-shaped ridgeline forms a natural boundary nearly 14 km long by two or three wide, extending to the southwest from the headwaters of the Aswa at the foot of the Labwor Hills [14]. The topography of the area effectively forces human movement around the southern slope, as an ecological gateway between the more ecologically fragile hills and rangelands of the northeast and the clement watered plains of the Lango region.

The prehistory of the study area, and the origin stories of the four cultures of relevance, originate in a complex and cyclical flow of human migrations undertaken in response to sustained periods of climatic stress; the names of famines from early times were passed down through centuries of oral tradition to be recorded during the mid-twentieth century, and validated into a chronology in the 1970s by early postcolonial scholarship drawing upon a record of Nile level measurements taken on the Nilometer on Rodah Island at Cairo, beginning in the Medieval era [26]. The historian’s task of dating the early history of present-day cultures is easier where there are king-lists, which are frequent among the more structured Bantu societies, including Buganda—but are also a feature of the Lwo cultural family, which arose in a ‘cradleland’ along the Bahr el Ghazal region of the Nile basin in present-day South Sudan [13, 26, 27].

While Lango culture is situated alongside the Acholi neighbors from whom they appropriated most of their language, they share a common historical origin with the Teso, as indicated by a remnant of distinctly Ateker-origin words—important nouns—remaining in Lango, including plant names and bodily organs (*e.g., ****emany*** for liver). Lango history traces its descent primarily back to the Jie clan of the Karimojong, from which likewise descended the Teso—but at some remote point, the Lango gave up or drastically adapted their ancestral Ateker-based language in to adopt a Lwo dialect. Uzoigwe [28] considers the Lwo language “a very simple one” and speculates that although Lango origins may have been predominantly Ateker-speaking, the relatively simplicity of the Lwo tongue was likely a factor in the Lango adoption of a language common to their Acholi neighbors. In linguistic terms, as compared to the Ateker, the Lwo language has relative lack of syllabic complexity, and is of shallow orthographic depth [29, 30], apparently contributing to its wide adoption as a simple and effective *lingua franca* within the study area.

### Cultural conceptions of health and illness

Extant within study area are culturally-specific explanatory models of health and disease, inclusive of 'folk etiologies' by which a given ailment may be related to internal states as well as external influences, in which the traditional healer serves as an intermediary between the patient and unseen causative agents both biotic and intangible [31, 32]. While the traditional healer relies on the bioactivity of plant-based medicines, her therapeutic portfolio may also involve purely ritual interventions to which are consigned illnesses or conditions ascribed to executive influences originating in the spirit world.

Central to the traditional religions of the Lango, Acholi and Ethur is the Lwo concept of ***Jok*** (***Juok*** in leb Thur) as supreme being, the lexical root from which is derived ***jogi*** (plural of the small-j ***jok***) as a generic name for individual spirits—including those particular to a clan or line of descent, those of known ancestors, and those of "unknown persons and dangerous beasts"—all of whom might possess a victim as a causative agent of affliction [33–38]. Likewise derived from the ***Jok*** root is the ***ajoka*** (Lango), ***ajwaka*** (Acholi) or ***ajuoga*** (Ethur)—a seer or diviner and healer who is initiated into knowledge of medicinal plants, and of the unseen influences which may cause physical or mental illness and may be propitiated by ritual (***etogo***) involving the individual, and at times the community (the latter category including chronic children's illnesses, impotence and eye disease) [39, 40]. In the words of the Acholi poet Okot p'Bitek, the healer is "a consultant psychiatrist, chemist and priest combined. He administered medicines which effected a cure and gave psychological treatment to patients who needed it" [38], including psychic ailments associated with social anxiety [41].

Writing a century ago, Driberg [33] describes an advanced Lango anatomical terminology (in which, for example, the hypogastrium or lower belly is named differently in men and women)—but laments the insufficiency of Lango medical nomenclature, in which ***etoku*** signifies yaws, eczema and sarcoma; ***angwal*** both paralysis and synovitis (synovial inflammation), and ***aola*** all manner of complaints of the throat, chest or lung involving cough. Indigestion was identified as "a much-dreaded complaint" then widely believed to be transmissible, and Driberg found the Lango "baffled" by all forms of veterinary illnesses. Atypically for a man of his era, Driberg credited the ***ajoka*** as being "endowed with wide knowledge and a scientific mind [as well as] primarily the physician of the soul… [with] an intimate knowledge of the use of herbs and plants for medicinal purposes above and beyond the popular remedies known to the vulgar, and certain operations are only performed by him, as for instance sucking the pus from boils" [33].

In opposition to the healer stands the wizard or ***ajok*** (***ajwak***) who delves in sorcery; as 'night-dancers' these wizards (who may be operating consciously or unconsciously) serve as agents of jealousy—attributed by the Ethur with causing complications in childbirth, elephantiasis and other disorders [42]. As causative agents of strife and illness, Lango culture recognizes a shade (***tipu***) originating in a person, and of non-human 'winds' (***yamo***)—elemental entities similar to the tiny beings of other cultures, which carry out the malign intentions of a ***tipu*** gone bad. In diagnosing occult ailments, chronic conditions with severe symptoms which can be traced to a known social conflict are the domain of shades, while passing ailments of less severe symptomology which cannot be traced to a social conflict are the domain of the winds [40]. Of the ***jok*** manifestations as diseases with a known ritual cure, the known and established endemic diseases were ascribed to the local ***Jok Lango***, but others to the foreign ***Jok Nam*** (god of the lake), ruler of diseases thought to originate from the Bantu peoples to the southwest [34].

Following Driberg by a generation, the anthropologist Hayley usefully classified Lango medical conditions into three broad categories, comprising inevitable diseases of childhood which also afflict adults; unexplained illnesses of a purely biotic origin (***two***), and those ascribed to a manifestation of Jok power, of which the latter included "psychic disturbances and virulent diseases such as plague"—***Jok*** (or a ***jok***) being identified as the cause as well as the potential cure of such afflictions [37]. Hayley was struck by the fact that epilepsy (***ekwinkwin***) was explicitly recognized as being of a purely natural origin (at the time, untreatable) and thus relegated to the second, ***two*** category and not the third—underlining the fact that not all types of unexplained medical phenomena were automatically ascribed to occult influence in traditional society [37].

By comparison, Teso culture has many names for the supreme being, including ***Akuj*** ('the one above,' associated with the sun), prime mover of all things good and evil; the healing god ***Ejokit*** (as 'the good'), and ***Edeke***—the 'god of calamity' (or vengeance) and a bringer of sickness, plague, and veneral disease [43, 44]. The latter name was also known among the Jie from which the Iteso descended [45]. It has been suggested that all events, including illnesses, have "a primary cause, Akuj, and a secondary cause, created agencies;" as a result, according to one traditional healer interviewed in 1979, it is unwise to rely on only one type of treatment for sickness [44].

Like the Lwo ***ajoka***, the Teso ***emurwon*** ('diviner-prophet') works with ethnomedicinal cures and with ***ajokin*** (pure spirits) to counter the activity of ***acudan*** (the witch or sorcerer) and malign intentions of the elemental spirits ***iboro*** (plural of ***ibore***), corresponding to the Lwo ***yamo***, which can be distinguished by an otherworldly smell, and are said to be active when the finger-millet is ripening [44]. Like the Lwo ***ajoka***, the Iteso ***emurwon*** receives their healing vocation from the spirit world (often after surviving a serious illness) but their phytomedicinal knowledge is transmitted from elder practitioners, and the institution is thought to predate the migration of the Iteso from their original homeland among the Jie [44, 45]. Like the ***ajoka***, the ***emurwon*** is both a healer of diseases both physical and spiritual, and an intermediary between the departed and the living—treating afflictions caused by spirit possession both in the individual and within the broader community.

Given its cosmopolitan ethnic and historical origins, it may be reasonable to suggest that these disparate lines of cultural descent and influence may have provided the Lango with a relative diversity of ethnomedical traditions as compared to the other three cultures. At the outset of the study, it was supposed that some evidence of shared origins might be discerned in the commonality of medicinal plant use along the Lango cultural interfaces with the Acholi and Teso in particular.

### Data collection

At the time of data collection, the continuity of traditional ways within the project area were reflected in the persistence of decentralized social and cultural institutions based around local (***jago***) chiefs and clan elders, particularly in the work-groups organized locally, in which agricultural labor was shared by neighbors on a rotating basis. These groups, known in leb Lango as ***wang tic*** (‘burn the work’) are often named after a vernacular proverb (e.g., the group name ***orib cing*** for ‘joined hands’—resonant of the proverb ‘many hands make light work’). These widely dispersed groups are constitute a social infrastructure which supported relief distribution during a series of droughts and crop failures in the 1980s, and provided access to revolving microcredit loans in support of rural livelihoods during the 1990s. Largely representative of the communities in which they are situated, with broad representation by women and the elderly in particular, these groups provide a forum for the exchange of ideas, making accessible the holders of traditional knowledge.

**Fig. 1:**
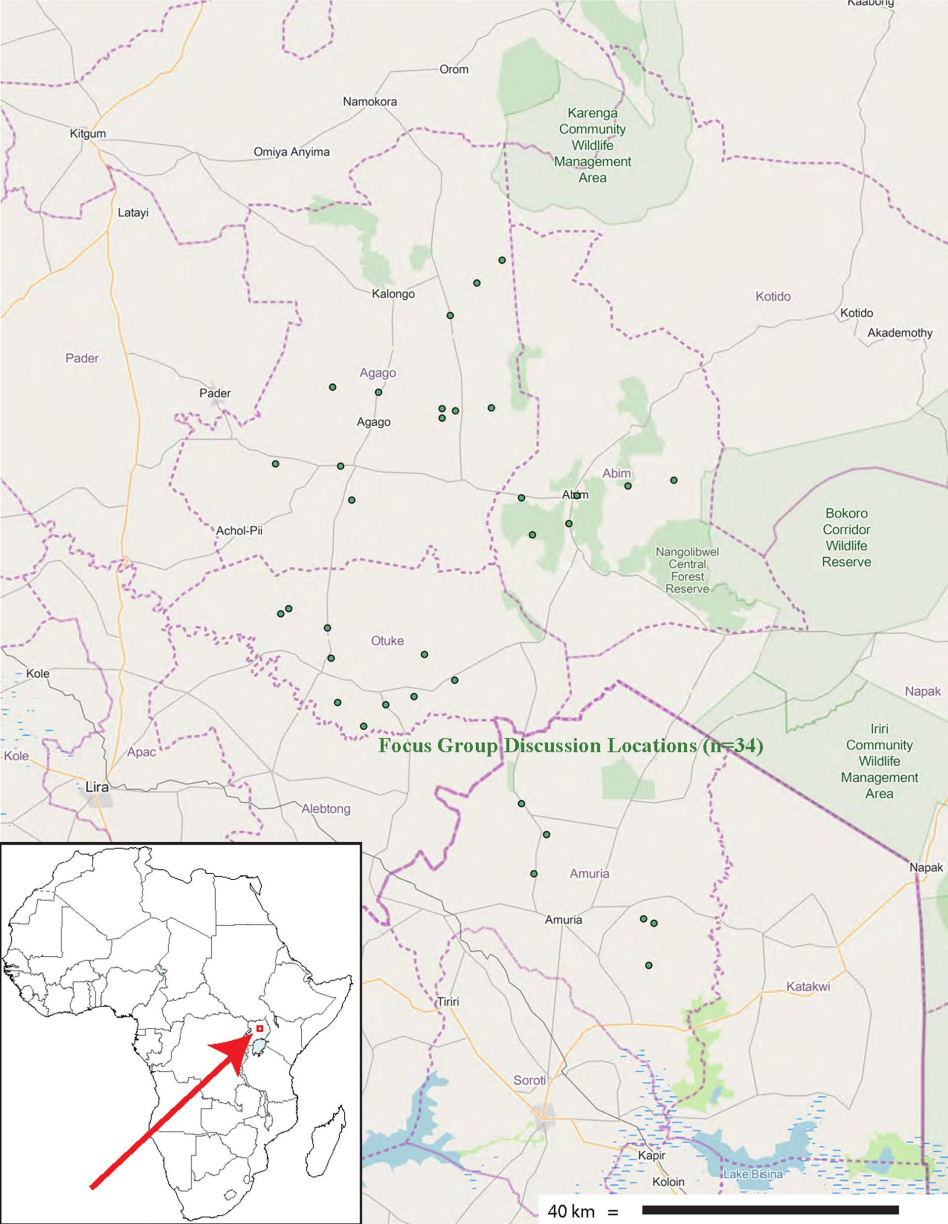
The study area

In order to establish prior informed consent among participating communities and individuals, introductory open meetings were convened at the community level over a period of several years, leading to a more focused set of community-based focus group discussions on plant use (Fig. 1), followed by a series of individual interviews on specific taxa, from which respondents holding specialized knowledge of plant use were engaged on basis of self-selection through purposive sampling, resulting in a de facto sample of 112 respondents, each of whom contributed one or more botanical specimens and use reports.

During 1999 and 2000, interviews on medicinal plant use were conducted with 112 subject matter specialists, each providing an herbarium specimen of a medicinal plant corresponding to one or more use reports, and describing the methods of harvest, processing and administration (Table 1). Herbarium samples were prepared by project staff and were subsequently presented to the Makerere University Herbarium (MHU) for identification and storage. Herbarium voucher codes for each specimen are provided by plant family and individual taxon in Table 2.

**Table 1:** The study area by district, respondents and herbarium vouchers

District	Culture	Taxa	URs	Informants	Men	Women	Herbarium vouchers		Women
				All			All	Men	
Otuke	Lango	55	92	**39**	30	9	**68**	58	10
Agago	Acholi	29	46	**22**	16	6	**35**	22	13
Amuria	Teso	60	118	**40**	28	12	**74**	58	16
Abim	Ethur	16	24	**11**	8	3	**17**	11	6
		**108**	**280**	**112**	**82**	**30**	**194**	**149**	**45**

Bold formatting is used to convey total figures where a parameter is disaggregated and at the bottom of a column

**Table 2:** All data summary by taxon and use report

Taxon	RI	Vernacular (Language)	Voucher No	Part*	Indications	Method
*Amaranthaceae*						
*Aerva lanata* (L.) Juss	**0.14**	Yat Dobo (A)	SA499	R	leprosy	powdered root mixed with tea or beer, taken orally
*Amaranthus spinosus* L	**0.36**	Akwata (T)	SA168	A	cough	aqueous extraction of leaves, stems and flowers taken orally
			SA168	A	headache	aqueous extraction of leaves, stems and flowers taken orally
			SA168	A	malaria	aqueous extraction of leaves, stems and flowers taken orally
*Celosia leptostachya* Benth	**0.14**	Ochwobteng (E)	SA415	R	vomiting	root paste mixed with water, taken orally
*Anacardiaceae*						
*Ozoroa insignis* Delile	**0.43**	Imuturu, Anino (L)	DN404	R	diarrhea	roots crushed and squeezed, the resulting juice taken orally
		Anino (E)	SA399	R	diarrhea	roots used to treat diarrhea
		Emuturu/Etiling (T)	SA480	R	headache	root paste diluted with water
*Sclerocarya birrea* Hochst	**0.36**	Titigo (A)	LA19	L, F	scorpion	leaves and fruit are crushed and applied to an incision
			SA480	R	pneumonia	root paste diluted with water
		Ejikai (T)	CE29	R	measles	root paste diluted with water and taken orally
			LA19	B, R	stomach	bark and root paste dilution taken orally
*Searsia pyroides* (Burch.) Moffett	**0.29**	Awaya, Awaca (L)	SA218	Br	dental	branch used as tooth-brush
		Awaya me witeta (L)	SA218	L	measles	leaves used to clean the tongue of someone infected with measles
			DN414	L	toothache	boiled leaves used for dressing
*Annonaceae*						
*Annona senegalensis* Pers	**0.64**	Obwolo (A, L)	SA21	B	diarrhea	bark taken orally
			SA223	R	anxiety (vet)	diluted root paste used to wash the animal
			SA21	R	diarrhea	bark paste dilution taken orally
			SA259	R	diarrhea	root paste dilution taken orally
			LA9	R	diarrhea	root paste dilution taken orally
			LA9	R	snakebite	root is consumed as antivenom
			SA120	R	wound	root paste applied topically
*Apiaceae*						
*Steganotaenia araliacea* Hochst	**0.21**	Olwiro (E)	SA413	R	snakebite	root paste used to treat snakebite 'by drinking' (dilution taken orally)
			SA413	R	snakebite	root paste used to treat snakebite 'by cutting' (applied to an incision)
*Apocynaceae*						
*Cynanchum insipidum* (E.Mey.) Liede & Khanum	**0.14**	Okura, Okuru (E)	SA432	R	cough	root paste taken orally
*Carissa spinarum* L	**0.14**	Emuriei, Emuriet (T)	SA461	R	measles	root paste mixed with *Strychnos innocua* (eturukuku)
*Pergularia daemia* (Forssk.) Chiov	**0.14**	Okuro (E)	SA429	R	unspecified	roots 'used for medicine'
*Araliaceae*						
*Cussonia arborea* Hochst. ex A.Rich	**0.36**	Ibucibuc (L)	PO26	L	oedema	boiled leaves used to dress swollen knees
			SA366	R	lameness	boiled roots used to treat 'the lame part of a person'
			PO26	R	pain	root paste administered to the skin for pains
*Arecaceae*						
*Phoenix reclinata* Jacq	**0.14**	Tit, Otit (L)	SA378	L, R	vomiting	boiled with *Indigofera confusa* (awee) leaves, into to a green decoction taken orally
*Asparagaceae*						
*Asparagus buchananii* Baker	**0.21**	Esigala Kiru (T)	SA463	R	cardiac	root paste diluted with water, taken orally
			SA463	R	STD	root paste diluted with water, taken orally
*Asparagus flagellaris* Baker	**0.43**	Ogudu (L)	SA217	F	eye	fruits eaten to prevent future eye problems
		Obudi (E)	SA423	F	eye	fruits used to treat eye problems
		Esigirikiru (T)	SA181	R	measles	root paste (mixed with Acinikoku herb), taken orally
			SA181	R	placenta	root paste (mixed with acinikoku leaf) taken orally to expel placenta (fast acting)
*Asphodelaceae*						
*Aloe volkensii* Engl	**0.14**	Tebakori (L)	SA292	L	cough	leaves and roots mixed with shea butter to reduce bitterness, taken orally
*Asteraceae*						
*Aspilia africana* (Pers.) C.D.Adams	**0.14**	Idudu Yikulu (L)	SA317	R	placenta	root paste decoction in warm water taken orally to speed expulsion of the placenta
*Baccharoides adoensis* (Sch.Bip. ex Walp.) H.Rob	**0.29**	Abwori, Labwori (A)	LA6	L	wound	boiled leaves used to dress wounds
			SA503	L	wound	boiled leaves used to dress wounds
			LA6	R	constipation	root decoction used as enema to treat constipation in children
*Berkheya spekeana* Oliv	**0.14**	Elem (T)	SA175	R	worms	dried root powder mixed with water, taken orally
*Chrysanthellum americanum* Vatke	**0.14**	Otibolok (T)	SA171	Fl	wound	paste of leaves, stems and flowers used on wounds as topical antiseptic
*Dicoma sessiliflora* Harv	**0.14**	Ocoko Lac (L)	SA216	R	urinary	root paste diluted with water, taken orally for urinary infections, particularly in children
*Echinops amplexicaulis* Oliv	**0.29**	Elem (T)	SA160	R	hydrocele	(globe thistle) roots and fruits pounded; decoction drunk for 'swollen testicles' (hydrocele)
			SA160	R	measles	(globe thistle) roots and fruits pounded; decoction drunk for measles
			SA160	R	stomach	(globe thistle) roots and fruits pounded; decoction drunk for stomach pain
*Erigeron floribundus* (Kunth) Sch.Bip	**0.21**	Odiltong (L)	SA323	L	wound	boiled leaves for dressing wounds
		Okwaras (T)	SA483	L	wound	leaf paste mixed with water (Teso) or boiled leaf decoction (Teso) used as topical antiseptic 'like iodine'—on fresh or old wounds
*Schkuhria pinnata* (Lam.) Kuntze ex Thell	**0.21**	Kilorokwin (T)	SA481	L	headache	leaf paste decoction taken orally
			SA481	L	malaria	leaf paste decoction taken orally
			SA169	L	unspecified	paste of young leaves and stem taken orally
*Synedrella nodiflora* Gaertn	**0.14**	Ebitikiliok T)	SA491	L	dental	paste of tender young leaves applied to infant gums to prevent 'false tooth'
*Vernonia perrottetii* Sch.Bip. ex Walp	**0.14**	Etimbine (T)	SA490	L	Eye	leaf decoction applied to eye to treat eye infection
*Bignoniaceae*						
*Kigelia africana* (Lam.) Benth	**0.50**	Yago (L)	DN435	F	Wound	Fruit decoction applied topically to wound
			SA306	F	Wound	Fruit decoction applied topically to wound
		Edodoi (T)	CE37	L	Eye (vet)	Leaf ash used to treat eye ailments in cattle
			CE37	R	Debility	Root paste as tonic (against 'general body weakness')
			CE37	St	Mumps	Culturally believed' that knocking one's head on the tree treats mumps
*Boraginaceae*						
*Trichodesma zeylanicum* (Burm.f.) R.Br	**0.14**	Agilo (L)	SA365	St	placenta	'uprooted and the ends are cut, washed and mixed with water to treat the after-birth '
			SA365	St	placenta (vet)	'uprooted and the ends are cut, washed and mixed with water to treat the after-birth of animal'
*Burseraceae*						
*Commiphora africana* (A.Rich.) Endl	**0.21**	Eledit (T)	SA352	R	cough	root paste decoction, mixed with *Zanthoxylum chalybeum* (eusuk), taken orally
			SA352	R	measles	root paste decoction, mixed with *Zanthoxylum chalybeum* (eusuk), taken orally
*Cannabaceae*						
*Cannabis sativa* L.	**0.14**	Jai (L)	SA294	L	anorexia (vet)	pounded with salt, fed to treat lack of appetite in animals (goats and sheep)
			SA294	L	diarrhea (vet)	fed to treat diarrhea in animals (goats and sheep)
*Capparaceae*						
*Capparis fascicularis* DC	**0.14**	Yat Yit (A)	SA497	R	ear	root paste decoction applied to ear canal
*Cleomaceae*						
*Cleome gynandra* L.	**0.29**	Akeo (L, E)	SA204	R	ear	root paste decoction applied to ear canal
			SA405	R	eye	root paste decoction applied to eye
*Colchicaceae*						
*Gloriosa superba* L	**0.29**	Yat Ania (L)	SA300	Bu	anemia	dilute paste of bulb given to children to treat anemia
			SA572	R	pneumonia	root paste diluted, used to treat pneumonia
*Combretaceae*						
*Combretum adenogonium* Steud. ex A.Rich	**0.43**	Meng, Omeng (L)	SA329	L	cough	paste of young leaves taken orally for cough (mixed with water for treating children)
		Anwanga (A)	LA3	L	dislocation	tender leaves used for dressing a dislocation
			LA3	L	fracture	tender leaves used for dressing a fracture
			SA377	R	diarrhea	dilute root paste taken orally
			SA377	R	diarrhea (vet)	dilute root paste also used to treat diarrhea in animals
*Combretum collinum* Fresen	**0.50**	Ekulony (T)	CE27	L	cough	young leaves eaten as a cough treatment
		Odugu (L)	DN393	R	diarrhea	root paste mixed with water (hot or cold), taken orally
			SA471	R	diarrhea	root paste mixed with water, taken orally
		Odugu (A)	LA23	R	diarrhea	root paste mixed with water, taken orally
			LA23	S	wound	seed paste used as topical disinfectant on wounds
*Combretum molle* R.Br. ex G.Don	**0.14**	Ekworo (T)	CE11	L	cough	young leaves eaten as a cough treatment
*Terminalia mollis* M.A.Lawson	**0.14**	Opok (L)	SA376	L	cough	young leaves are chewed for cough
*Convolvulaceae*						
*Lepistemon owariense* (P. Beauv.) Hallier f	**0.79**	Akeng (L)	CE51	R	diarrhea	filtered root decoction taken orally
		Esor, Esoro (T)	SA586	R	hernia	diluted root paste taken orally
			SA586	R	malaria	diluted root paste taken orally
			CE51	R	measles	filtered root decoction taken orally
			CE51	R	muscular	filtered root decoction taken orally for muscle pain, pull
			SA179	R	placenta (vet)	Given to animals to facilitate placenta drop in premature birth
			SA179	R	pregnancy (vet)	root decoction given to animals that give premature kids
			SA179	R	stomach	filtered root decoction mixed with roots of *Acacia sieberiana*, taken orally
			SA355	R	worms	root paste mixed with water taken orally
			SA355	R	worms	root paste mixed with water mixed with salt, taken orally (for animal)
*Cucurbitaceae*						
*Momordica foetida* Schumach	**0.21**	Bomo (L)	SA570	R	cough	root paste diluted with water, taken orally
			SA570	R	stomach	root paste diluted with water, taken orally for stomach ache
*Zehneria scabra* Sond	**0.14**	Ebomo (T)	SA157	L	measles	leaf paste smeared around neck
			SA157	R	measles	root decoction taken orally
*Ebenaceae*						
*Euclea racemosa* L. subsp. *schimperi* (A.DC.) F.White	**0.57**	Imuk (L)	DN423	B	stomach	bark treats stomach ailments
		Emus (T)	DN423	R	dental	root paste used to clean the teeth
		Omush (E)	SA327	R	dental worms	root paste used to clean the teeth 'against worms'
			SA178	R	STD	root paste dried, later mixed with water and administered orally for gonorrhea 'and others'
			SA419	R	toothache	root paste applied to tooth
			SA419	R	yellow fever	root paste decoction taken orally
*Euphorbiaceae*						
*Acalypha brachiata* Krauss	**0.29**	Ayilayila (L)	SA166	R	diarrhea	roots dried and powdered, taken orally; water added for treatment of children
		Ecolong-Obiel (T)	SA219	R	pregnancy	root paste mixed with water, administered orally to prevent miscarriage ('given to one who is aborting')
*Euphorbia bicompacta* Bruyns	**0.29**	Iburka (L)	PO20	La	cataract (vet)	latex applied to eye for cataracts in cattle
			SA332	La	swelling	stem latex applied topically to reduce swelling
*Jatropha curcas* L	**0.57**	Ejumula (T)	CE47	L	sprain	leaves used to massage sprains, and 'breasts which are sick'
			CE47	R	fainting	root paste mixed with boiling water, then given orally 'to one who has fainted'
			SA465	R	poison	root paste mixed with water as antidote to poisoning
			SA465	St	eye	stem (?) juice applied to eye
			SA465	St	eye (vet)	stem (?) juice used to treat eye problems in animals
*Fabaceae*						
*Abrus precatorius* L	**0.14**	Ewinywiny (T)	SA493	L	impotence	leaf paste decoction taken orally
*Albizia amara* (Roxb.) B.Boivin	**0.29**	Kikwaa (A)	LA27	L	wound	leaf paste applied to wounds as topical disinfectant
		Thino (E)	SA410	R	dysentery	root decoction taken orally
*Albizia coriaria* Welw. ex Oliv	**0.43**	Itek (L)	SA335	B	STD	bark decoction taken orally
		Etekwa (T)	DN476	B	stomach	boiled bark paste taken orally for stomach pain
			LA15	R	measles	Root paste taken orally
*Albizia schimperiana* Oliv	**0.14**	Ibata-atar (L)	DN475	R	cataract	root paste diluted slightly, applied to eye to treat cataracts
			DN475	R	cataract (vet)	root paste diluted slightly, applied to eye to treat cataracts in livestock
*Chamaecrista nigricans* (Vahl) Greene	**0.43**	Ajebi (L)	SA308	L	chest pain	leaf paste decoction taken orally
		Acwaa (A)	SA308	L	cough	leaf paste decoction taken orally
		Epeduru (T)	SA163	L	cough	leaf paste decoction taken orally
			SA163	L	sore throat	leaves chewed for sore throat
			SA252	L	worms	leaf paste taken orally as an anthelmintic—dried leaf paste can be stored for a year
			SA308	L	wound	leaf paste applied to wounds as topical antiseptic
			SA308	L	wound	leaf paste applied to wounds as topical antiseptic in animals
			SA252	L	wound	leaf paste applied to wounds as topical antiseptic
			SA252	L	wound	leaf paste applied to wounds as topical antiseptic in animals
*Erythrina abyssinica* DC	**0.14**	Iwilakot (L)	DN457	R	paralysis	root paste and crushed bark diluted with water, applied to incisions in the skin for paralysis
*Indigofera arrecta* Hochst. ex A.Rich	**0.14**	Awee (L)	SA378	L	vomiting	Leaves boiled with *Phoenix reclinata* (tit) leaves to a green decoction taken orally; root paste also used for this purpose
			SA378	R	vomiting	root paste taken orally as an anti-emetic (to treat vomiting)
*Indigofera emarginella* Steud. ex A.Rich	**0.57**	Teletele (A)	SA486	R	impotence	root paste boiled in milk and 'other herbs', given over 2–3 days to treat impotence
		Ederut, Esuroi (T)	SA176	R	STD	root paste dried (can keep up to 2 months), mixed with *Strychnos innocua* (akwalakwala) roots to treat gonorrhea
			SA240	R	stomach	root paste given to children for stomach ache
			SA281	R	stomach	root paste given to children for stomach ache
			SA176	R	urinary	root paste dried (can keep up to 2 months), mixed with *Strychnos innocua* roots as diuretic
*Neorautanenia mitis* (A.Rich.) Verdc	**0.14**	Ekulac (T)	SA343	R	ectoparasite (vet)	veterinary: root paste applied to skin as acaricide, to treat ticks and lice in dogs
*Philenoptera laxiflora* (Guill. & Perr.) Roberty	**0.14**	Olwedo (L)	SA295	R	stomach	diluted root paste taken orally to treat stomach-ache
*Piliostigma thonningii* (Schumach.) Milne-Redh	**0.29**	Ogali (A)	SA340	Fi	wound	fiber applied to injury as bandage
		Epapai (T)	LA24	R	diarrhea	root paste mixed with water, taken orally
*Pleurolobus gangeticus* (L.) J.St.-Hil. ex H.Ohashi & K.Ohashi	**0.14**	Yat Aola (L)	SA312	R	cough	roots chewed directly, to treat cough in adult
			SA312	R	cough	root paste boiled in oil, taken orally to treat child's cough
*Rhynchosia hirta* (Andrews) Meikle & Verdc	**0.14**	Agaba (L)	SA370	R	psychosis	root paste burnt, smoke inhaled by patient 'to chase devils' (antispsychotic)
*Rhynchosia* sp. Lour	**0.21**	Aremo-remo (L)	SA358	R	diarrhea	root paste mixed with water, taken orally
		Ookot (T)	SA466	R	diarrhea	root paste mixed with water, taken orally
*Senegalia polycantha* (Willd.) Seigler & Ebinger	**0.14**	Okutu-agwe (L)	SA311	R	swelling	roots used to wash a patient to reduce swelling (to treat a patient with ***auraur***, or 'swollen body')
*Senna siamea* (Lam.) H.S.Irwin & Barneby	**0.36**	Gacia (A)	CE39	L	measles	paste of young leaves diluted, taken orally
		Egasia (T)	CE39	R	hepatitis	root paste diluted, taken orally
			LA13	R	STD	root paste diluted, taken orally
*Senna singueana *(Delile) Lock	**0.71**	Akwaradok (L)	SA502	R	boils	root paste applied to skin
		Akwara Dok (A)	SA575	R	diarrhea	root paste diluted, taken orally
		Engerengerei (T)	SA338	R	leprosy	root paste mixed with water, taken orally
			SA502	R	yellow fever	root paste taken orally
			SA338	R	pregnancy	root paste mixed with water, taken orally to treat abdominal pain during pregnancy
			DN416	R	toothache	root paste mixed with water, taken orally
*Tamarindus indica* L	**0.43**	Cwaa (A, E)	SA406	F	cough	pulp 'juice' taken orally to treat cough
			LA20	F	measles	fruits are threshed in water, the liquid is then dropped in the ear, mouth and eyes of a child suffering from measles
			LA32	F	measles	boiled fruit taken orally
			SA406	F	measles	boiled fruit taken orally
			SA232	S	measles	seeds put in water and taken orally
*Vachellia amythethophylla* (Steud. ex A.Rich.) Kyal. & Boatwr	**0.29**	Eorotom (T)	SA165	R	cough	pounded root paste, dried or wet, mixed with *Acacia hockii* (Ekisim) and *Acalypha villicaulis* (Ecolong-obiel), taken orally (water added, for children)
			CE20	R	measles	root paste taken orally
*Vachellia drepanolobium* (Harms ex Y.Sjöstedt) P.J.H.Hurter	**0.21**	Ayelel (E)	SA424	R	diarrhea	root decoction taken orally
			SA424	R	measles	root decoction taken orally
*Vachellia hockii* (De Wild.) Seigler & Ebinger	**0.36**	Okutu-ryang (L)	CE18	Fl	lactation	flowers rubbed with little water on the breasts of a woman who has lost her baby, to stop the milk flow
		Ekisim (T)	CE18	Fl	measles	root decoction taken orally
			DN462	R	measles	roots roasted, decoction taken orally (residue discarded)
			SA167	R	measles	roots roasted, decoction taken orally (residue discarded)
*Vachellia sieberiana* (DC.) Kyal. & Boatwr	**0.29**	Etirir (T)	SA348	R	diarrhea	Root paste taken orally
			SA348	R	measles	Root paste taken orally
			CE66	R	measles	Root paste mixed with 'other herbs', taken orally
*Hypericaceae*						
*Psorospermum febrifugum* Spach	**0.43**	Amoo (L, A, T)	DN479	R	scabies	dried roots and bark mixed with shea butter, applied to skin
			SA150	R	scabies	dried roots and bark applied with shea butter
			SA559	R	scabies	dried roots and bark applied with shea butter
			CE3	R	tuberculosis	a decoction of the root bark is administered orally to treat tuberculosis
*Iridaceae*						
*Gladiolus dalenii* Van Geel	**0.29**	Alotcak, Ibworka (L)	SA304	Bu	cough	bulb chewed for cough
			SA304	Bu	debility	bulb chewed 'to treat any sickness' (general tonic)
			SA304	Bu	poison	bulb is is used 'for treating poison'
*Lamiaceae*						
*Hoslundia opposita* Vahl	**0.36**	Apurumur, Edudu (L)	SA211	R	epilepsy	root paste diluted, used to used to wash the head
		Tutu (A)	SA276	R	epilepsy	root paste used to treat epilepsy; applied to facial cuts, ears, nostrils and eyes, 'also wash the head and bum'; the paste can be dried and stored for later use
			SA206	R	unspecified	roots 'used as medicine'
*Leonotis nepetifolia* (L.) R.Br	**0.50**	Nyamtigo A)	SA182	L	cough	leaf paste diluted with water, taken orally to treat cough
		Ecika (T)	SA182	L	STD	leaf paste diluted with water, taken orally to treat gonorrhea ('it is mixed with ETIPET')
		Chukuchuku (E)	SA400	L	wound	leaves boiled and used to clean wounds
			SA273	R	measles	root paste decoction 'sprinkle[d] around the room where one with measles is'
			SA273	R	measles	root paste decoction taken orally
*Ocimum forskaolii* Benth	**0.43**	Mida (L)	SA420	L	eye	leaf decoction applied to eye to treat eye infection
		Emopim (T)	SA336	L	disinfection	resin from leaves used to clean hands;
		Mitho (E)	SA345	L	disinfection	resin from leaves used to clean hands;
			SA475	L	disinfection	resin from leaves used to clean hands;
*Rotheca myricoides* (Hochst.) Steane & Mabb	**0.43**	Okwero (L)	SA220	R	constipation	root paste decoction taken orally as laxative
		Ekweru (T)	CE67	R	eye (vet)	root fluid used as eye drops to treat eye problems in cattle
			SA220	R	pain	root paste decoction applied to incision (Lango)
			SA164	R	stomach	root paste mixed with *Carissa spinarium* and *acolong obiel,* diluted water and taken orally to treat stomach pains (Teso)
*Vitex doniana* Sweet	**0.14**	Owelo (L)	SA49	F	diarrhea	fruits taken orally
*Vitex madiensis* Oliv	**0.14**	Owelo Too (L)	PO5	R	measles	root paste administered orally to children
*Loganiaceae*						
*Strychnos innocua* Delile	**0.43**	Eturugugut,	SA159	F	debility	'you cannot get sick when you eat [the fruit]'
		Eturukuku (T)	SA461	R	measles	Root paste used to treat measles (mixed with *Carissa spinarium*)
			CE22	Br	dental	Small branches used as tooth-brush
*Loranthaceae*						
*Loranthus* sp. Jacq	**0.14**	Elit (T)	CE11	P	cough	taken orally
*Malvaceae*						
*Grewia mollis* Juss	**0.14**	Pogo (A)	LA1	R	diarrhea	root, water extract of bark taken orally to treat diarrhea in children
*Grewia villosa* Willd	**0.14**	Olukuru (A)	SA268	R	anemia	root decoction used to treat anemia in children ('it adds blood'); used to wash 'a child whose hair has turned brown'
*Sida ovata* Forssk	**0.14**	Idokoko (T)	SA145	R	stomach	dried root decoction (tea), taken orally to treat stomach ache
*Meliaceae*						
Pseudocedrela kotschyi Harms	**0.57**	Eputu, Iputo (L)	SA215	R	diarrhea	root paste mixed with water, taken orally
		Oput (A)	SA333	R	diarrhea	root paste mixed with water, taken orally
		Eputon (T)	LA22	R	diarrhea	root paste mixed with water, taken orally
			CE8	R	measles	root paste decoction taken orally
			DN397	R	STD	root paste 'mixed with warm/cold water', taken orally for treating *venereal* diseases
*Trichilia emetica* Vahl	**0.21**	Akwirakwir (L)	PO17	R	snakebite	root paste used to treat snakebite
			PO17	R	worms	root paste used to treat worms
*Menispermaceae*						
*Chasmanthera dependens* Hochst	**0.14**	Akeng (L)	SA296	T	pneumonia	tuber paste decoction taken orally
*Moraceae*						
*Ficus glumosa* Delile	**0.29**	Okworo (L)	LA4	B	diarrhea	water extract of bark taken orally
		Kworo (A)	DN386	Fi	postnatal	bark fiber used to tie the umbilical cord
*Ficus sycomorus* L.	**0.14**	Olam (A)	LA12	L	leprosy	leaves rubbed at the point of infection to treat leprosy
*Ficus vasta* Forssk	**0.14**	Pwoo (A)	LA5	B	diarrhea	bark paste diluted with water, taken orally
*Olacaceae*						
*Ximenia americana* L.	**0.14**	Elamai (T)	CE6	R	measles	root paste taken orally
*Oxalidaceae*						
*Oxalis corniculata* L.	**0.14**	Yat Leny (L)	SA45	L	heartburn	leaves boiled, taken orally to treat heartburn
*Phyllanthaceae*						
*Bridelia scleroneura* Müll.Arg	**0.29**	Orweco (L)	DN395	B	diarrhea	bark taken orally
		Erieco (T)	CE19	R	diarrhea	roots mixed with 'other herbs' to treat diarrhea
			CE19	St	dental	small branches used as tooth-brush
*Flueggea virosa* (Roxb. ex Willd.) Royle	**0.29**	Elakas (T)	SA341	R	headache	roots used to treat headache
			CE55	R	measles	root paste diluted with hot water, taken orally
			SA341	R	measles	root paste diluted with hot water, taken orally
*Phyllanthus amarus* Schumach. & Thonn	**0.14**	Elilit (T)	SA492	L	Cough	leaf paste diluted with water, taken orally
*Poaceae*						
*Cymbopogon citratus* Stapf	**0.21**	Enyait (T)	SA468	R	Delivery	root paste taken orally to ease childbirth
			SA468	R	Measles	root paste used to treat measles
*Perotis patens* Gand	**0.14**	Epeero (T)	SA476	L	Cough	young, tender leaves chewed for cough
*Polygalaceae*						
*Securidaca longepedunculata* Fresen	**0.29**	Ilila (L)	SA330	R	Headache	root decoction applied to cuts made in forehead, as analgesic to treat headache
		Ediol (T)	SA417	R	Headache	root decoction used as analgesic to treat headache
		Lilia (E)	CE48	R	Unspecified	root bark decoction (may be dried for storage)
*Rubiaceae*						
*Fadogia glabberima* Hiern	**0.14**	Acet Dyel (L)	SA369	L	Hernia	leaves mixed with *Pavetta crassipes* (anyango) leaves, pounded and mixed with water
*Gardenia ternifolia* Schumach. & Thonn	**0.29**	Odwong (A)	SA271	R	Snakebite	root paste used to treat snakebite
		Ekore, Ekoroi (T)	CE50	R	Measles	root paste diluted with hot water, taken orally
*Pavetta crassipes* K.Schum	**0.43**	Anyango (L)	SA153	L	Cough	leaf tea taken orally
		Egwapet, Egwapit (T)	SA153	L	Cough (vet)	leaf tea administered orally
			SA369	L	Hernia	leaves mixed with *Fadogia glaberima* leaves, pounded and mixed with water
			SA369	L	Measles	leaf decoction used to bathe a patient
			SA589	L	Measles	fresh leaves boiled, the 'tea' used to clean the mouth
*Sarcocephalus latifolius *(Sm.) E.A.Bruce	**0.71**	Ebele, Ibele (L)	SA282	R	Hernia	root paste taken orally to treat hernia
		Ibele (A)	SA375	R	hernia	root paste mixed with water or beer and drunk
		Eutudolei (T)	SA246	R	Pain	root paste applied to incisions, or taken orally, as analgesic 'for any pains'
			DN452	R	Prolapse	root paste used to treat rectal prolapse [also believed to be a result of cutting the tree]
			SA462	R	STD	root paste mixed with water, taken orally to treat STDs in general
			SA462	R	Wwelling	root paste mixed with water, taken orally for 'swollen thighs'
			SA462	R	Urinary	root paste mixed with water, taken orally as a diuretic
*Spermacoce princeae* (K.Schum.) Verdc	**0.29**	Etakalar,	SA177	L	Ringworm	leaf juice or paste applied to skin to treat ringworm
		Etakalere (T)	SA474	L	Ringworm	dried leaf paste (powder) mixed with oil, used to treat ringworm
			SA583	L	Dermatitis	leaf juice, or dried leaf decoction, applied to skin to treat 'skin rashes'
*Rutaceae*						
*Harrisonia abyssinica* Oliv	**0.86**	Akere, Okutu Akeri (L)	PO1	L	Cough	young leaves chewed to treat cough
		Opedo (A)	LA28	L	Cough	young leaves chewed to treat cough
		Ekerei, Ekeroi (T)	SA326	L	Postnatal	young leaves are put into a sauce of pigeon peas, and given to a woman who has just given birth
			CE56	L	Stings	leaves used as first aid on wasp stings
			LA28	R	Diarrhea	root paste decoction taken orally to treat diarrhea in children
			SA322	R	Stomach	root paste taken orally to treat stomach ache
			CE56	R	Stomach	root paste diluted with warm water, taken orally to treat stomach ache
			CE56	R	Measles	root paste diluted with warm water, taken orally
			SA494	R	Measles	root paste mixed with *Carissa spinarium*, taken orally
*Vepris nobilis* (Delile) Mziray	**0.14**	Ekudep (T)	CE49	L	Measles	young leaves are pounded and steamed, the vapor inhaled to treat measles in children
			CE49	R	Measles	root decoction (pounded root soaked in boiling water, and sieved), taken orally
*Zanthoxylum chalybeum* Engl	**0.57**	Eusuk (T)	CE23	B	Measles	both bark and roots may be dried, pounded / crushed and mixed with water, taken orally
		Okore (E)	CE23	R	Measles	both bark and roots may be dried, pounded / crushed and mixed with water, taken orally
			SA357	F	Cough	hot water infusion of fruits and/or roots administered orally to treat cough, in animals
			SA357	R	Cough	hot water infusion of fruits and/or roots taken orally
			SA352	R	Cough	root paste decoction, mixed with *Commiphora africana*, taken orally
			CE23	S	Cough	seeds crushed with water and taken orally
			SA396	R	Toothache	root paste applied to tooth to treat toothache
*Sapindaceae*						
Sapindus saponaria L	**0.14**	Enyama (T)	CE40	B	worms (vet)	roots and bark administered in feed as anthelmintic for cattle
*Sapotaceae*						
*Vitellaria paradoxa* C.F.Gaertn. subsp. *nilotica* (Kotschy) A.N.Henry, Chithra & N.C.Nair	**0.14**	Yaa (A)	SA258	B	diarrhea	bark powder taken orally
*Solanaceae*						
*Physalis minima *L	**0.14**	Etaagole (T)	SA485	L	postpartum	leaf paste applied to umbilical cord of newborn
*Solanum incanum* L	**0.71**	Ocokocok (L)	SA310	F	ear	boiled fruit decoction applied to ear canal to treat earache
		Ocok (A)	SA173	F	eye	fruit juice applied to eye to treat infection
			SA409	F	eye	fruit juice applied to eye to treat eye infection
		Etulelut (T)	SA173	F	eye (vet)	fruit juice applied to eye to treat veterinary eye infection
		Ococok (E)	SA409	F	eye (vet)	fruit juice applied to eye to treat veterinary eye infection
			SA409	F	wound	fruit juice applied to wound as topical disinfectant
			LA29	F	toothache	fruit juice mixed with parrafin to make drops applied to tooth
			SA356	R	diarrhea	root paste diluted with water, taken orally
			SA409	R	diarrhea	root paste diluted with water, taken orally
			SA409	R	diarrhea	root paste diluted with water and applied as an enema to treat diarrhea
			SA173	R	stomach	root paste diluted with water, taken orally for stomach ache
*Vitaceae*						
*Cissus cornifolia* Planch	**0.29**	Amuru Gweno (L)	SA314	R	swelling	root paste applied to skin to treat boils (reduce swelling)
*Cyphostemma adenocaule* Desc. ex Wild & R.B.Drumm	**0.14**	Emoros (T)	SA144	L	debility	leaves consumed in sauce 'you cannot get sick after eating'
*Zygophyllaceae*						
*Balanites aegyptiaca* (L.) Delile	**0.14**	Ecomai (T)	CE13	La	eye (vet)	'Latex' (resin or gum) is 'used as eye drops for bulls that are hurt'

Bold formatting is used for derived statistics (RI numbers)

^*^ Key to Part(s) used: A—aerial parts; B – bark; Br – branch; Bu – bulb; F – fruit; Fi – fiber; Fl – flower; L – leaf; La – latex; P – whole plant; R – root; S – seed; St—stem; T—tuber

Concurrently with the data collection, an inventory of woody species and its size-class distribution was undertaken on a set of fixed and variable plots in the current districts of Otuke and Amuria [46], with an indication of overall frequency of woody species by size-class within these plots. Unfortunately, this work could not be extended to the current districts of Agago and Abim due to increasing insecurity including insurgent attacks on civilian targets within the study area at the time of data collection [47].

### Data analysis

In August of 2022, and again in June 2023, the taxa list was updated according to the current botanical nomenclature. Based on the updated and current list of taxa, use reports were triangulated by three rounds of secondary research, beginning with a review of relevant ethnobotanical use reports, 13 of which were proximate to but outside of the current study area.

Standard reference books were also consulted, including J.O. Kowaro’s *Medicinal plants of East Africa* [48] and the five volumes of *The Useful Plants of West Tropical Africa*, H.M. Burkhill’s 1980–1990s revision of the original 1937 compendium by Dalziel [49–53]. A third reference resource of note is the online Prelude Medicinal Plants Database maintained by the Belgian AfricaMuseum (formerly Royal Museum for Central Africa), which yielded 30 corroborating use reports from 21 sources which could not be obtained directly [54]. A second database, PROSEA (Plant Resources of South-East Asia), was accessible during the first round of secondary research [55] and then went offline, but has been restored as of the time of publication.

In a second round of secondary research, the therapeutic profile of each taxon was assembled from the results of published pharmacological studies (mostly using in vitro and animal models), and from phytochemical analyses with a focus on bioactive compounds (either explicitly implicated, or potentially relevant). A third round of research surveyed the documented bioactivity and pathways of the specific molecules identified as being present in a particular taxon.

### Comparative indices

The purpose of the study being to assemble an inventory of medicinal use applications among the four cultures, the interviewers did not request comprehensive or ranked lists of medicinal plants from each respondent, but use reports were based on the botanical specimens which were accessible to each respondent at the time the interview was conducted. Use of plant specimens as a focal point for structured interviews has been referred to as the ‘plant interview’ method of ethnobotanical research [56].

Data from the interviews were correlated in the aggregate to determine the relative importance of each taxon using the Relative Importance Index (RI), developed for ranking of medicinal plants by their pharmacological properties, and by the body systems affected [57]. The RI is a simple measure of the diversity of uses of a given taxon, without factoring in the number of informants [58], and without regard to any ranking of relative importance of each taxon by respondents.

$${\mathrm{RI}}_{s}= \frac{{\mathrm{PH}}_{s(\mathrm{max})}+{\mathrm{BS}}_{s(\mathrm{max})}}{2}$$

where PH_*s*_(max) is obtained by dividing the normalized number of pharmacological properties attributed the species (PH_*s*_) by the maximum value in all medicinal species surveyed [PH_*s*_(max) = PH_*s*_/max (PH)], and BS_*s*_(max) is the normalized number of body systems affected by the species (BS) divided by the maximum number of body systems affected by all medicinal species cited [57].

Although some subsequent authors notably including De Albuquerque et al. have limited their calculations to published clinical data [59], the RI is used here to assess the relative importance of the cited taxa according to the totality of use reports for each taxon, not disregarding out of hand those use reports for which no prior published reports can be found.

In order to test the operational hypothesis, the Jaccard Index of similarity (JI) was first calculated as an indicator of relative degree of confluence (similarity) in the inventory of plants used as medicine among and between the four cultures of the study area [60], considering the ratio of species used by multiple cultures as compared to those cited by only one culture, without regard to commonality of specific use application.

$$\mathrm{JI}=\frac{a}{(a+b+c)}$$

where *a* is the number of taxa cited by both populations, *b* is the number of taxa cited only by the first of the two populations, and *c* the number of taxa cited by the second of the two populations [61].

Finally, the similarity of specific medicinal usage between the four cultures was assessed using Rahman’s Similarity Index (RSI), which provides a percentage of common use applications for each taxon between two populations.

$$\mathrm{RSI}={\left( \frac{d}{a+b+c-d} \right)}\mathrm{x} 100$$

where *a* is the number of taxa uniquely cited within the first population, *b* is the number of species unique to the second population, *c* is the number of taxa common to both populations for all uses, and *d* is the number of common species used to treat similar ailments by both populations [62, 63].

## Results

Table 1 presents a summary of interview data and herbarium specimens collected from 112 respondents in 67 locations within the four districts, languages and cultures of the study area. The 112 key informant interviews yielded 289 medicinal use reports for 194 herbarium specimens used as medicine, subsequently identified as 108 taxa from 44 botanical families, of which just 12 families account for more than two thirds of all use reports. Fabaceae was by far the most cited plant family, at 20% of cited taxa and 21% of all total use reports.

Table 2 presents 280 medicinal use reports describing 263 different use applications for the treatment of 62 maladies or conditions (use categories), including the plant part used, preparation and administration and method of use, and the Relative Importance ranking of each taxon. Of the Relative Importance figures presented in Table 2, 13 taxa were ranked at 50% or higher, while 5 taxa were ranked above 70% (Table 3).

**Table 3:** Taxa with Relative Importance Index (RI) values of 0.5 or greater

Family	Taxon	RI	URs	Cultures
Rutaceae	*Harrisonia abyssinica*	**0.86**	9	3
Convolvulaceae	*Lepistemon owariense*	**0.79**	10	2
Rubiaceae	*Sarcocephalus latifolius*	**0.71**	7	3
Solanaceae	*Solanum incanum*	**0.71**	11	4
Fabaceae	*Senna singueana*	**0.71**	6	3
Annonaceae	*Annona senegalensis*	**0.64**	6	2
Meliaceae	*Pseudocedrela kotschyi*	**0.57**	5	3
Rutaceae	*Zanthoxylum chalybeum*	**0.57**	7	2
Ebenaceae	*Euclearacemosa subsp. schimperi*	**0.57**	6	3
Fabaceae	*Indigofera emarginella*	**0.57**	5	2
Combretaceae	*Combretum collinum*	**0.50**	5	3
Bignoniaceae	*Kigelia africana*	**0.50**	5	2
Lamiaceae	*Leonotis nepetifolia*	**0.50**	5	3

Bold formatting is used for derived statistics (RI numbers)

**Fig. 2:**
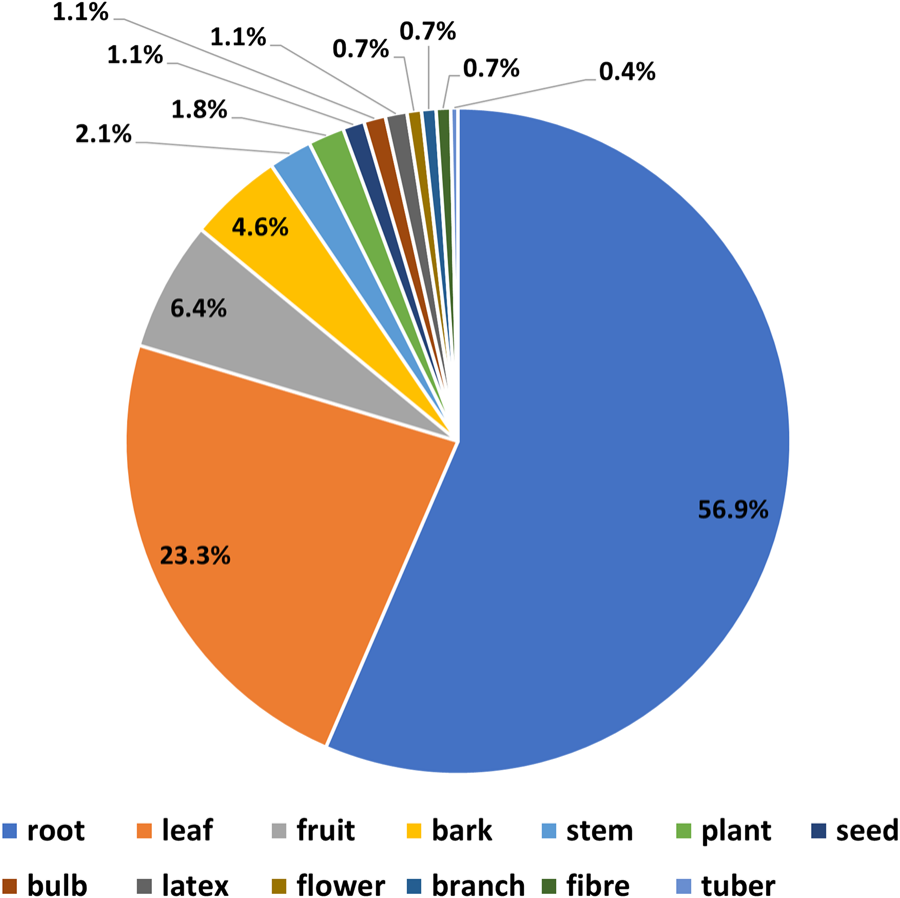
Plant parts used as medicine by proportion of Use Reports

Plant parts used as medicine (Fig. 2) indicated a strong preference for the root, comprising about 60% of all use reports, followed by leaf (over 23%) in most cases involving dilution or decoction of the macerated root paste taken orally. Notable exceptions to this method of administration include those plant compounds which are applied topically to the skin (*Cussonia arborea* to treat pain and swelling, *Cissus cornifolia* for boils) or to cuts made in the skin (*Rotheca myricoides* and *Sarcocephalus latifolius* for pain, *Erythrina abyssinica* for paralysis, *Steganotaenia araliacea* to treat snakebite, *Sclerocarya birrea* for scorpion sting).

**Fig. 3:**
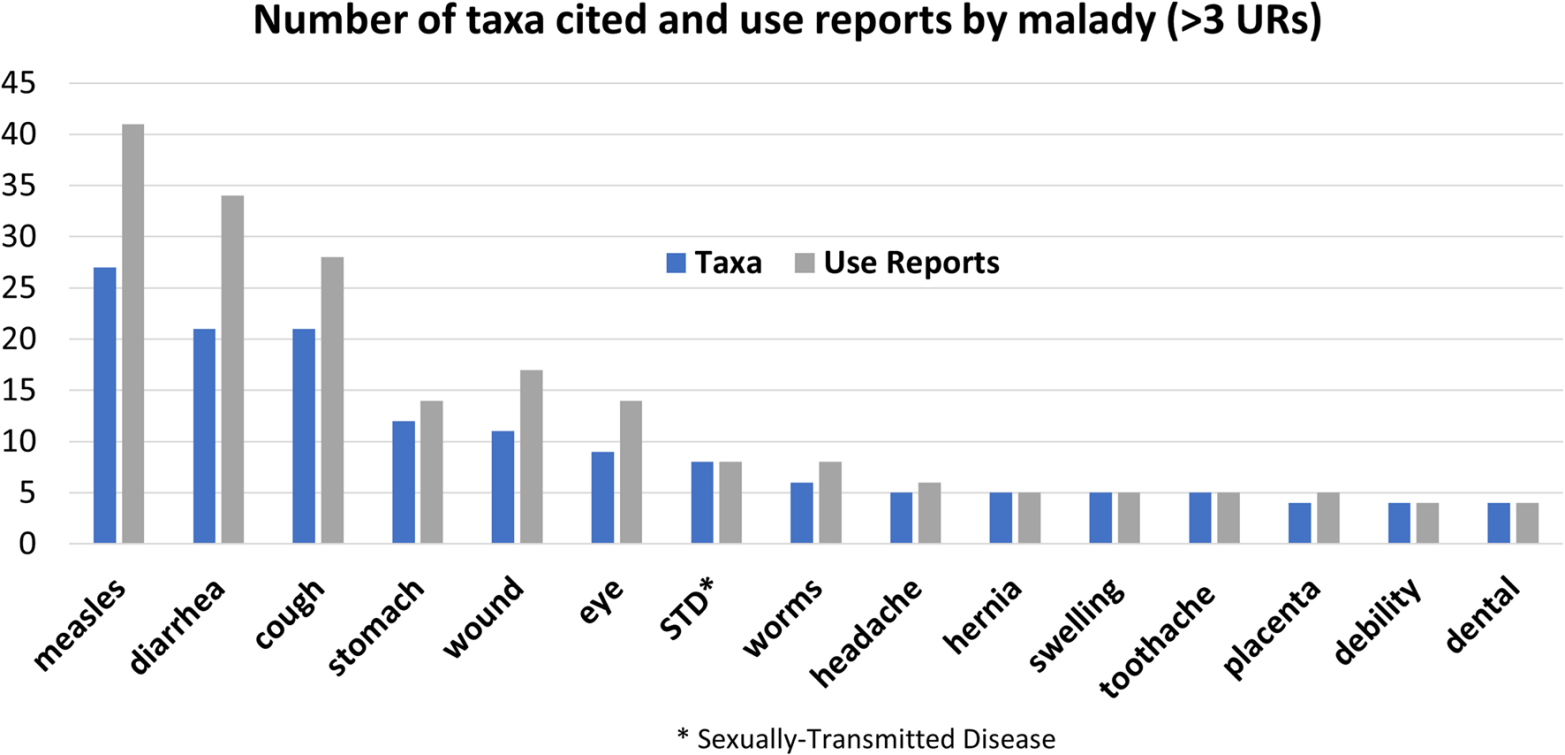
Ranking of maladies by number of Use Reports

The number of cited taxa and use reports by malady (Fig. 3) indicates an overall ranking frequency of three conditions—measles, diarrhea, and cough, all at above 20 taxa cited per condition, followed by stomach problems and wounds (all above 10 taxa cited), eye problems, sexually-transmitted diseases, and worms (above 5 taxa cited), and a long tail of eight maladies ranging between 5 and 3 taxa cited. With 41 use reports involving 27 taxa, treatment of measles was by far the most cited medicinal application, closely followed by measles and cough, and a second level of prevalence for stomach problems, wounds, and eye infections.

**Fig. 4:**
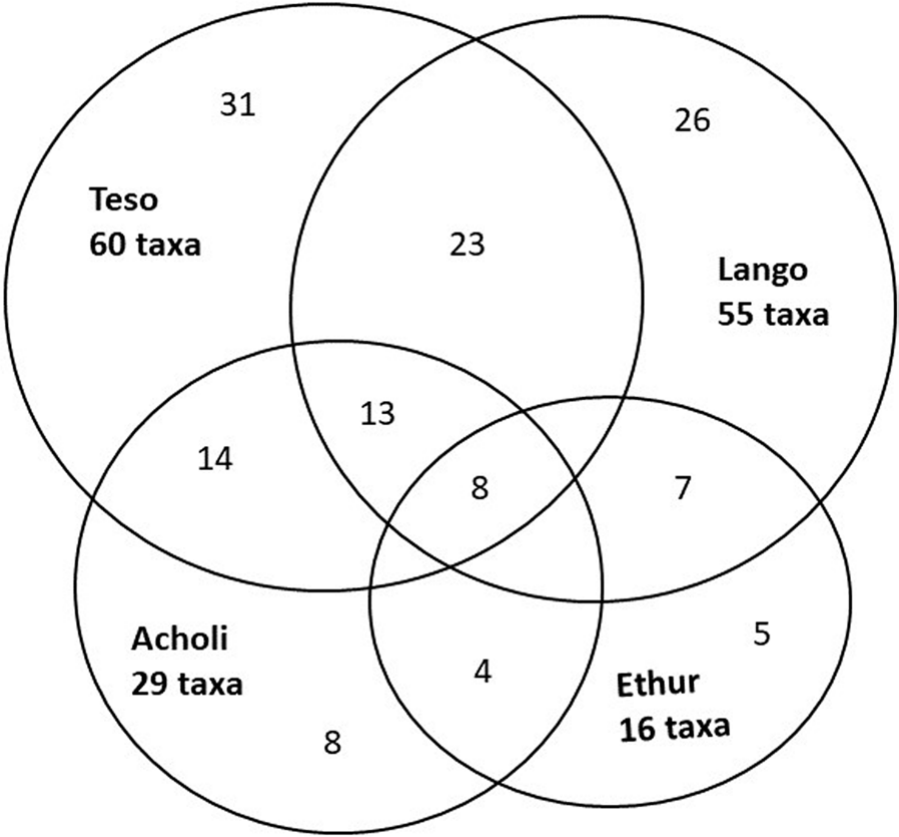
Medicinal taxa cited by culture and interface (*n* = 108)

Figure 4 and Table 4 show how the cited taxa are distributed by culture, including commonality of plant use along the six cultural interfaces, while Table 5 indicates the commonality of specific medicinal use application between the four cultures, indicating the strongest commonality between the Lango and Acholi cultures (united by markedly similar languages), by a factor of two over the Lango and Teso cultural interface, which represents shared historical origins, but no longer a common language.

**Table 4:** Medicinal taxa cited by culture, and in common between cultures

	Lango	Acholi	Teso	Ethur
	**55**	**29**	**60**	**16**
Lango	26	13	23	7
Acholi		8	14	4
Teso			31	8
Ethur				5

Bold figures represent all medicinal taxa cited by culture, underlined figures are taxa cited solely by that culture

**Table 5:** Similarity of taxa and medicinal use by culture

Cultural Interface	Shared taxa	Shared indications	Jaccard Index (%)	Rahman's SI (%)
Lango/Acholi	13	7	**12.1**	**20.5**
Lango/Teso	23	7	**21.5**	**9.6**
Lango/Ethur	7	3	**6.5**	**8.6**
Acholi/Ethur	4	1	**3.7**	**6.3**
Teso/Ethur	8	2	**7.5**	**4.8**
Acholi/Teso	14	1	**13.1**	**3.9**

Bold formatting is used for derived statistics (JI and Rahman's SI)

Of the 108 taxa cited across the study, 49 use reports cited 15 species in 18 identical use applications for 10 maladies shared between two or more cultures, of which only one species use application was shared between three cultures—*Psorospermum febrifugum* root for treatment of scabies (Table 6).

**Table 6:** Commonality of use application by malady and cultural interface

Indication	All		Taxon	Cultural Interface	Common
	URs	Taxa			URs
Measles	40	27	*Vachellia hockii*	Teso and Thur	3
			*Tamarindus indica*	Acholi and Thur	2
Diarrhea	33	21	*Combretum collinum*	Lango and Acholi	3
			*Ozoroa insignis*	Lango and Thur	2
			*Pseudocedrela kotschyi*	Lango and Acholi	3
			*Rhynchosia* sp.	Lango and Teso	2
			*Solanum incanum*	Teso and Thur	3
Cough	28	21	*Harrisonia abyssinica*	Lango and Acholi	2
			*Chamaecrista nigricans*	Lango and Teso	2
Stomach	14	12	*Harrisonia abyssinica*	Lango and Teso	2
Wound	17	11	*Chamaecrista nigricans*	Lango and Acholi	4
			*Conyza floribunda*	Lango and Teso	2
Eye	14	9	*Solanum incanum*	Teso and Thur	4
			*Asparagus flagellaris*	Lango and Thur	2
Headache	6	5	*Securidaca longipedunculata*	Lango and Thur	2
Scabies	3	1	*Psorospermum febrifugum*	Lango, Acholi and Teso	3
Hygiene	3	1	*Ocimum forsskaolii*	Lango and Teso	3
Epilepsy	2	1	*Hoslundia opposita*	Lango and Acholi	2

Not included in this analysis of cultural similarity of use were those use reports involving different plant parts of the same species used to treat the same malady*, e.g., Bridelia scleroneura* bark used in Lango to treat diarrhea, while the root is used in Teso for that purpose. One species, *Solanum incanum*, was cited by respondents of Teso and Ethur for unrelated ailments (root for diarrhea, fruit for eye infection).

## Discussion

### Context of historical studies

As provided in the supplemental file, review of the secondary research yielded 403 citations from 139 ethnobotanical publications, including full or partial matches to species/genus, plant part and administration by indication (malady or condition), providing full or partial corroboration of 193 (73%) of the 263 reported use applications. Of the 70 use applications which were not found in any earlier ethnobotanical reports, 16 (22%) were found to be possibly supported by secondary data from in vitro and in *vivo studies*, including those involving compounds reported to be present in the respective taxon or genus.

Of the 263 use applications by taxon listed in Table 2, 191 are at least partially corroborated by one or more earlier ethnobotanical studies drawn from across the entire Africa region. Indeed, several of the cited taxa have been identified among the earliest medicinal plant remains recovered by archeological studies in northern Nigeria, including *Vitex* spp. dating back to c. 2800–2450 BP, and *Bridelia scleroneura, Pavetta* sp**.** and *Sarcocephalus latifolius* dated earlier than 800 CE [64].

No earlier mention can be found within the earlier ethnobotanical literature of 72 of the 263 medicinal applications in the current study (about 27% all use reports obtained. One notable example is *Lepistemon owariense* (Convolvulaceae), with an RI value of 79% based on four reported pharmacological properties affecting seven body systems, for which no earlier mention has been found. Other use applications undocumented in the earlier literature include seven taxa used to treat measles (*Albizia coriaria*, *Gardenia ternifolia.*, *Leonotis nepetifolia, Pavetta crassipes, Strychnos innocua*, *Vitex madiensis* and *Zanthoxylum chalybeum*); three as anti-emetic (*Celosia leptostachya*, *Indigofera arrecta, Phoenix reclinata*); *Dicoma sessiliflora* for urinary problems, and *Vernonia perrottetii* for eye problems.

Of the apparently novel use applications, eight have some association with bioactivity in pharmacological (in vitro or animal model) or phytochemical studies, including antiviral compounds in *Commiphora Africana*, *Chrysanthellum americanum* and *Vernonia perrottetii*; antibacterial compounds in *Albizia amara* and *Synedrella nodiflora*; anti-inflammatory compounds in *Vachellia hockii* and *Senegalia polyacantha*, diuretic compounds in *Sarcocephalus latifolius*, and antimicrobial, anti-inflammatory, analgesic and antipyretic compounds in *Physalis minima*, the leaf-paste of which is applied to the umbilical cord of the new-born infant.

**Fig. 5:**
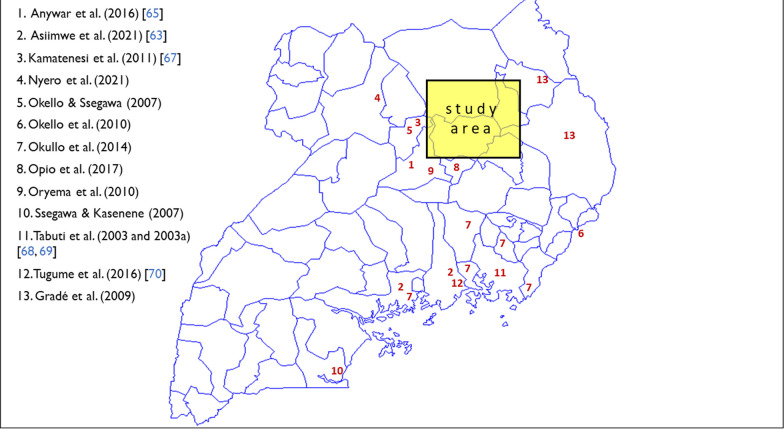
Geographic context of the Uganda studies

Figure 5 provides a geographic and historical context of the ethnopharmacological studies undertaken within Uganda, indicating that no earlier data were collected within the study area.

Of the earlier Ugandan studies mapped in Fig. 5, most accounts were limited to a specific ailment or condition [53, 65, 66] or a specific locality and culture [51, 52, 67–70].

### Floristic and ecological context

From an ecological perspective, the number of cited taxa and use reports was compiled by plant family with intention to compare the findings with earlier studies, and with what is known of the floristic composition of the study area. Unfortunately, the earlier data (notably including the species lists compiled by Langdale-Brown et al*.* [14]) do not indicate relative abundance or frequency of occurrence, and the inventory and size-class distribution data collected on fixed and variable plots within the study area concurrently with data collection, some results of which were presented in an earlier paper [71], include only woody and not herbaceous species. Nonetheless, the degree to which Fabaceae have been selected for medicinal use stands in contrast to their natural occurrence—comprising over 24% of all medicinal use reports, as compared to the woody species inventory data, which places the family at just 4.2% of woody species by basal area in Otuke, and 18.6% by species composition in Amuria.

### Folk taxonomy and medicinal use categories

Table 2 is organized by family, and some commonality of use is visually evident within particular genera and families—examples including the remedies for cough and diarrhea within the Combretaceae, wound care among the Compositae, treatment for diarrhea in Moraceae, hernia among the Rubiaceae, and measles and cough among the Rutaceae.

Table 2 provides the common vernacular phytonyms for each taxon cited according to each of the cultures citing it for a medicinal use. Classification systems within the vernacular nomenclature reflect emic dimensions of cultural meaning [32] by which plants are classified according to folk systematics [72]. In general, the vernacular phytonyms presented here differ between cultures in the interpretive accessibility of information held within them, partly a function of the linguistic structures of the vernacular languages in which they are recorded.

Among the Lwo phytonyms, such linguistic qualifiers include adjectives referring to morphological characters, with the adjectives white, black and red (***atar***, ***achol*** and ***rema***) commonly used to distinguish plant species within a genus or family. Taxonomically, the generic name for a group of species formerly classified together under the genus *Acacia* is ***okutu***, of which *Vachellia sieberiana* is known as ***okutu atar***, or white acacia; the genus *Terminalia* is called ***opok*** (of which *T. mollis* is ***opok acol***, black terminalia), and in the genus *Albizia* (***ibata***), *A. schimperiana* (***ibata atar***) is distinguished from another, non-medicinal species known as ***ibata achol***.

Other Lwo adjectives and descriptive nouns are likewise used as qualifiers in the vernacular nomenclature to distinguish related species within a genus, such as *Amaranthus caudatus* L. (***ocobo***), from which *A. graecizans* L. is distinguished as local by the qualifier ‘Lango’ (as ***ocobo Lango***), in the same manner that ash filtrate is called ‘local salt’ (***kado Lango***). Similarly, the cultivated *Cleome gynandra* L. (***akeo***) is distinguished from *C. monophylla* L. as ***akeo Jok***, in which the qualifier may signify its wild occurrence, or other properties.

While some Lwo plant names have a clear meaning for which a basis is not evident (for example, ***kongo ogwal-ogwal*** or ‘frog’s beer’ for *Physalis minima*), the basis for others is more directly related to the pharmacological activities attributed to the plants. Notable examples here include ***ocoko lac*** for *Dicoma sessiliflora*, a treatment for urinary infections, in which ***ocoko*** stands for the name of the plant, while the suffix ‘***lac***’ signifies urine, thus indicating its application.

Perhaps the most pharmacologically significant example is the qualifier ‘***yat***’. Defined by Kokwaro and Johns as “a general term for shrub, tree or medicine” [73], these three meanings are listed separately by other sources [74, 75], the latter separating its meanings as referring to a tree (or wood), to medicine or to an herb. In each such case, ***yat*** is followed by a second noun, often indicating a malady or condition to which the plant can be applied as a remedy, and thus indicative of its primary pharmacological property according to the current or past therapeutic application.

Examples of such nomenclature provided in Table 2 include *Aerva lanata*, ***yat dobo*** (or ‘leprosy medicine’ in leb Acholi); *Pleurolobus gangeticus*, ***yat aola*** (cough medicine); *Oxalis corniculata*, ***yat leny*** (heartburn medicine). Another example is *Gloriosa superba*.—***yat ania***, referring to its use in treatment of pneumonia (despite the notoriously extreme colchicine toxicity of the plant), and the tuber of an unidentified creeper called ***yat cak*** (milk medicine), credited with increasing milk production in humans and in livestock. Although no identification could be made on basis of the specimen provided, the taxonomic description and reported morphology of the plant are consistent with *Dioscorea* L. species, of which several are known to contain estrogenic compounds [76], and nine are present within the study area [77]. Estrogen is known to stimulate development of the mammary ducts, and, in association with progesterone, to stimulate proliferation of secretory tissues [78].

Examples of Ateso plant names which seem to imply their given therapeutic use may include *Vachellia hockii* (***ekisim***), the flowers of which are rubbed on the breasts (***ekisin***) of a mother who has lost her child to stop milk flow; *Pseudocedrela kotschyi* (***eputon***), the root of which is used to treat measles (***epuru***), and *Zanthoxylum chalybeum* (***eusuk***), various parts of which are used as a cough treatment—the word for lung given being ***euko*** [79]. By contrast, the name for *Rhynchosia* sp. (***ookot***) is strongly suggestive of blood (***aokot*** [80]), but is only cited here as a diarrhea treatment in Teso. This again may imply a former medicinal use no longer in practice within the respondent communities of the study area.

### Data interpretation in the epidemiological context

From the etic perspective, results are consistent with the documented epidemiology of the study area. Together accounting for over 36% of use reports, the findings around the three major maladies—measles, diarrhea and cough—largely reflect the historical prevalence of these conditions among the respondent communities [81]. The national measles vaccination coverage rate in 2002 was just 74% [82], leaving over a quarter of all Ugandan children unprotected against measles; this number is likely to be considerably higher within the study area, given regional disparities in health-related outcomes and access and availability of treatment [83]. Bbaale [84] notes that rates of both diarrhea and acute respiratory infection are highest in the northern region, which he conjecturally linked to the history of civil conflict and displacement ending in 2008.

While measles was the malady most cited by number of taxa and number of combined use reports, this ranking is based specifically on the 27 use reports from Teso (Amuria District), the highest value (by a factor of two) of any malady by district. The highly contagious disease measles (rubeola) is caused by a single-stranded, negative sense RNA virus in the genus *Morbillivirus* of the family Paramyxoviridae, which also includes other parainfluenza viruses, *e.g.,* mumps, rubella, and respiratory syncytial virus (RSV); measles results in a wide variety of health complications including pneumonia, blindness and chronic neurological conditions—and, although largely preventable by vaccine, measles remains responsible for an estimated 100,000 deaths each year [85–89]). Given the limited coverage of measles vaccination, these figures underline the relevance of locally available ameliorative treatments to manage the symptoms of the disease [90].

While a wide variety of phytochemical compounds have been found to inhibit viral replication via various pathways [91, 92], very few compounds are specifically linked to inhibition of the measles virus itself. Recently identified as antiviral agents with therapeutic value [93, 94], non-flavonoid phenolic coumarins and their derivatives (including pyranocoumarins) have been found to inhibit measles virus replication [95]. Although the mechanism of action is not understood, coumarin-like compounds have been found to interfere with or inhibit the viral enzymes of other, single-stranded positive sense Ribonucleic acid (RNA) viruses including Human Immunodeficiency Virus (HIV) [85]. According to the compositional studies consulted, coumarins and coumarin derivatives have been reported as present in 9 of the 27 taxa cited by respondents as being used in treatment of measles (Table 2).

Diarrhea was the highest-ranked malady by taxon and use report in three of the four cultures (Lango, Acholi and Ethur), by a factor of two over the second-ranked malady in those cultures. Diarrhea is the second leading cause of death globally in children under five years old, and a leading cause of malnutrition in that age group; nearly 1.7 billion cases of childhood diarrheal disease result in an estimated 525,000 child deaths each year [96]. Ugandans suffer the highest mortality rate of children under 5 years old in the East Africa region [97], with diarrhea accounting for over 20% of child deaths, against a global average of 8.6% [98, 99]. At the time of the data collection, prevalence of diarrhea within the northern region was the highest in Uganda, at any moment affecting 29.3% of the population [100], while a 2018 study specific to Agago District found the proportion children under 5 suffering from diarrhea to be over 40% [98].

Causative organisms of acute diarrhea include viruses (notably *Rotavirus*), Gram-positive and Gram-negative strains of bacteria (notably *Shigella* and *Campylobacter* spp., *Escherichia coli* and *Staphylococcus aureus*), and protozoa (notably *Giardia lamblia*) [101]. Phytochemical compounds effective in treating diarrhea include polyphenol catechins and tannins [102, 103] and the flavonol quercetin [104], specifically through increased colonic water and electrolyte reabsorption [103].

Cough was ranked at second in terms of use reports in all for regions, including Teso with 16 use reports. Persistent cough in Uganda has been linked to household air pollution including exposure to indoor smoke from cooking and lighting [105], and to infection by human pathogens including the influenza virus, particularly during the rainy season [106] and the Gram-negative, aerobic, pathogenic, encapsulated coccobacillus *Bordetella pertussis*, responsible for an estimated 400,000 annual deaths, mostly of infants in developing countries [99]. Several other bacteria—most notably the Gram-positive *Streptococcus pneumoniae* but also the Gram-negative *Mycoplasma pneumoniae* and *Chlamydophila pneumoniae*—are implicated in infectious pneumonia, the leading cause of mortality among children under 5 in sub-Saharan Africa, accounting for 12.8% of all such deaths [98]. In Uganda, Acute Respiratory Infections (ARIs) remain the leading cause of childhood morbidity and mortality among under-five children [107].

Phytochemical compounds with documented therapeutic activity in the treatment of cough include polysaccharides, anthraquinones and their derivatives. An in vivo study of cats [108] found cough suppressive activity in polysaccharide compounds isolated from nine plant species. [109]. Another study found that anthraquinone derivatives ameliorate lung inflammatory response, an effect recently reviewed with particular reference to Aloe (Asphodelaceae), Senna (Fabaceae) and Rheum L. (Polygonaceae) species [110].

Of the taxa cited for treatment of cough, *Aloe volkensii* is conspicuously abundant in polysaccharides [111], with a phytochemical profile including anthraquinones and pre-anthraquinones [112], as well as alkaloids, saponins, glycosides, flavonoids, and tannins [113] [114], noting that antioxidant compounds have been associated with a protective effect in cough suppression [115, 116].

Other maladies and conditions for which fewer use reports were obtained and species cited are nonetheless highly significant in terms of their mortality or morbidity, notably including snakebite—responsible for an estimated 32,000 annual deaths across sub-Saharan Africa (a quarter of the global total), leaving as many as 100,000 survivors with permanent physical disabilities [117]. The health burden on survivors of snakebite may involve hemorrhage, tetanus, contractures (debilitating stiffening of muscle or connective tissue), myonecrosis (life-threatening muscle infection), scarring, and tissue inflammation that result from the bites [118], resulting in 5000–15,000 amputations annually [119].

Horse-derived antivenin sera comprise the sole medical treatment for snakebite—but these are highly perishable, are unable to prevent local tissue damage, can induce adverse reactions including anaphylactic shock, and are scarce and unaffordable in the rural areas where nearly all envenomation occurs [118]. A 2018 study decried a compound global "crisis" of poor antivenom quality, availability and reliability of supply [120], and other authors have questioned the clinical effectiveness of available products within the Africa region in particular, as many such antivenin products were developed specifically to treat envenomation by Asian snake species [117]. In northern Uganda, where more than a third of snakebite victims are younger than 18 years, antivenom supplies are insufficient even at hospitals for optimum treatment of envenomation according to WHO guidelines [121]. These interrelated factors underline the importance of locally available envenomation treatments.

Of the four taxa cited for use in treatment of snakebite, *Annona senegalensis* Pers. root for treatment of snakebite was corroborated nine times in the ethnobotanical reports, and has been assessed clinically against venom of puff adder *Bitis arietans* [122], black-necked spitting cobra *Naja nigricollis* [123, 124], and West African Carpet viper *Echis ocellatus* [125].

No clinical antivenom studies were found on *Gardenia ternifolia*, although a 2015 study observed anti-inflammatory cyclooxygenase-1 (COX-1) inhibitory activity in an unspecified ethanolic extract, noted as a pathway of relevance to mouse hind paw oedema induced by *Bothrops insularis* (Golden Lancehead pit viper) snake venom [126]. Two studies [127, 128] have evaluated the in vitro antioxidant activity of *G. ternifolia* leaf extract containing flavonoid aglycones.

Although *Steganotaenia araliacea* root is cited as a snakebite remedy in seven ethnobotanical sources (with another report for its leaves), no clinical studies nor phytochemical data could be found for the species. No clinical evidence of antivenom activity was found on *Trichilia emetica*, but compositional studies indicate the presence of compounds with activity along potential antivenom pathways, including limonoid triterpenoids in root bark with notable anti-inflammatory properties [129, 130], noting further that a 2007 study reported a neurotoxin blocking activity of the limonoid triterpenoid toosendanin [131]. The flavonoid glycosides found to be present in a *T. emetica* seed extract [132] have been found to inhibit the phospholipase A2 (PLA2)-II toxins associated with some snake venoms [133, 134].

Although malaria is evidently a malady of great significance in northern Uganda, and the leading cause of morbidity and mortality in Uganda [135], it was ranked very low in the data (with just two use reports in Teso, and one in Lango), possibly reflecting the availability of pharmaceutical treatments, which are widely sold across the region by local traders and at weekly rural markets. The vernacular name for *Schkuhria pinnata*, reported as being used to treat malaria, was recorded as ‘***kilorokwin***’ in Ateso—certainly not a word characteristic of that language, but phonetically identical in the local pronunciation to the antimalarial compound chloroquine (chloroquine phosphate)—long the first line of pharmaceutical defense against non-resistant *Plasmodium* infection.

The sole taxon cited by respondents (in both Lango and Acholi) as a treatment for epilepsy, *Hoslundia opposita*, is abundantly evidenced in both the ethnobotanical record and in clinical studies. Noting its use in treatment of convulsion and epilepsy, as well as vertigo and mental disturbance, the anticonvulsant (central nervous system depressant) activity of a chloroform root extract of *H. opposita* was assessed in an animal model at 60% protection against leptazol-induced convulsions, and the extract was credited with potentiating the phenobarbitone sleeping time with an anticonvulsant activity comparable to benzodiazepines [136]. A later study [137] confirmed these results with respect to in vitro γ-Aminobutyric acid (GABA)_A_-benzodiazepine receptor binding activity of an ethanolic *H. opposita* leaf extract.

Cited in 7 use applications across three body systems for an RI of 71%, the root paste of *Sarcocephalus latifolius* is applied to incisions, or taken orally, as analgesic 'for any pains’. This use application is corroborated by multiple ethnobotanical sources cited by Burkhill [49]. More recently, a highly controversial 2013 paper [138] claimed to have isolated from the root ( ±)cis-2-[(dimethylamino) methyl]-1-(3 methoxyphenyl) cyclohexanol—a morphine analog commonly known by its international non-proprietary name, tramadol. A 2016 study [139] followed up on this controversial finding with a comprehensive study of *S. latifolius* which identified multiple antinociceptive compounds, most notably indoloquinolizidine alkaloids to which they attributed antiplasmodial and antibacterial, as well as analgesic activities.

Beyond the purely medical conditions which can be cross-referenced to the clinical data, respondents cited indications for more clinically ambiguous conditions which cannot be identified with confidence, which presented interpretive challenges in terms of data triangulation, which are discussed in the limitations section below. Four taxa were cited with reference to use applications loosely corresponding to ‘debility’—interpreted by the author to reflect use of the specified extract as an immunostimulatory tonic. Among them, the ‘bulb’ (corm) of *Gladiolus dalenii* was said to be chewed ‘to treat any sickness,' while the root paste of *Kigelia africana* was indicated as treatment for ‘general body weakness’. Respondents likewise ascribed immunostimulatory activities in the leaves of *Cyphostemma adenocaule* and the fruit of *Strychnos innocua* (‘you cannot get sick after eating’).

Perhaps related to these applications are the two taxa more specifically cited for treatment of anemia—the bulb extract of *Gloriosa superba* (notoriously toxic due to its high levels of the cytotoxic alkaloid colchicine), and the root extract of *Grewia villosa*. The antihemolytic activity of G. superba extracts has been evaluated in in vitro studies [140, 141], while a 2010 study [142] reported in vitro antioxidant and in vivo hepatoprotective activity γ-lactones isolated from a stem extract of a species identified as '*Grewia tiliaefolia*' (presumably *G. tiliifolia* A.Rich., syn. *G. amicorum* Steud.).

In another ambiguous indication, the root paste of *Rhynchosia hirta* was reported to be burnt, the smoke inhaled by patient ‘to chase devils’—interpreted by the author as an antipsychotic treatment. While the ethnobotanical literature holds no corroboration of this usage, the presence of alkaloids and saponins in *Rhyncosia* species as been noted [143] noted, to which antipsychotic activities have been [144].

Other notable results which beg a degree of cultural interpretation include the reported use of *Synedrella nodiflora* in treatment of a condition called ‘false tooth’ or *ebino*. Although classified here as a dental condition, in fact there is no clinical basis to support a cultural belief, widespread within the region, that the eruption of primary canine teeth causes fever and diarrhea, often leading to removal of the emerging tooth buds using unsterilized instruments and without anesthesia or antiseptic [145]. Several years prior to the study, the author witnessed one such extraction within the Lango sub-region, effected with a sharpened bicycle spoke.

The belief in ‘false tooth’ has been explained by emergence of the primary canine teeth corresponding to the time of first exposure to a range of ailments including diarrhea, which may lead to dehydration which may make the tooth bud more pronounced in appearance [145]. Because complications following the extraction are linked to increased morbidity and mortality, the use of an herbal treatment in lieu of traditional extraction (rightly termed ‘infant oral mutilation’ [146] may be considered a positive development.

### Limitations

According to other contemporaneous data sets obtained in the study area in parallel to this study, the results presented here seem to have under-reported the actual use of some taxa—most notably including *Vitellaria paradoxa* subspecies *nilotica*, around which the broader study of on-farm biodiversity was oriented. In addition to a biodiversity inventory and size class distribution of woodland species within the project area, results of which were published in an earlier paper [71], the project facilitated applied research on the cultural values of the tree which yielded 105 use reports specific to the medicinal uses of the tree in Lango and Teso in particular, compiled in a graduate thesis by a project intern [147].

In corroboration of the sole use report obtained for that species in this study, treatment of diarrhea in the Bratcher data was mentioned in 12 use reports focused on the roots of the tree, and 10 use reports on the bark, and a single use report on the leaves, with treatment of stomach ache similarly reported for roots (7 use reports), bark (5), shea butter (2) leaves (1) and seed. Of the 105 use reports in that report, 32 focused on topical use of shea butter for ‘smearing’ of new-born infants to protect the skin, also reflecting a cultural belief that the practice facilitates growth and strength (in the words of one respondent, ‘so they'll sleep nicely and grow fast with smooth skin’), with 7 use reports on the same application for children after bathing, 7 on use of shea butter to treat wounds, 4 for cough, 2 for stomachache, 2 to treat the cut umbilical cord, 2 to treat dry or sun-burnt skin, 2 for massage for sickness or dislocations, and one each for use of the liquid shea butter as a decongestant, to treat swelling (or infection), rashes, scabies, for skin care in pregnancy, for removing splinters or things from ear, as an emetic, and to treat measles and tuberculosis. Single use reports were collected for *V. paradoxa* roots cited for treatment of sexually-transmitted infections and worms, and flowers for measles, the leaf latex to treat fresh wounds, and the dross (seed matter remaining after shea butter extraction) in veterinary medicine to treat a chick's eyes and ears. These findings indicate that a methodology not strictly based on the availability of botanical specimens may have yielded a higher number of use reports by respondents.

The results presented here are bounded by limitations imposed by the study design, as well as the circumstances of its implementation and analysis. Although the literature around best ethnobotanical methods and their theoretical basis has been prolific in the intervening years since the data were collected [148], at the time of the data collection these methods were largely nascent [56]. A fundamental limitation of the study is that it was not originally intended for publication, but as a baseline and diagnostic study of on-farm biodiversity within the four communities (and cultures) served by an integrated conservation and development project. According to the parallel data set described above, this focus seems to have led to compensatory under-reporting on that species in particular—further suggesting that the data presented here are less than exhaustive in scope.

Presenting additional challenges to its analysis, data presented here were collected using two different data collection instruments over the course of the study, requiring careful reading of all original entries by the author. The original form used as a guide in the first key informant interviews initially allocated limited space for data collection, inconveniently listing five specimens over two paper pages—a layout requiring much back-and forth by the interviewer, and limited opportunity for elaboration on the part of the respondent. A second instrument was subsequently developed to provide a dedicated full page to each taxon, which allowed for supplementary notes to be added by the researcher to accommodate additional details of cultural context and practice.

Because participation was voluntary and sampling based on self-selection, some holders of relevant knowledge may have opted out, or not shared all available information on the maladies and conditions for which data were collected; we cannot know what was withheld or lost.

Another notable limitation is the rigidity of the study protocol, limiting data collection to taxa for which botanical voucher specimens could be collected and successfully identified by the national herbarium at Makerere University, in line with standard ethnobotanical practice [149, 150]. Thus, the timing of each interview would have limited the availability of the required botanical material, as would the seasonal morphology of the taxon, and its form; in the earlier publication on food plants [71] cited above, this requirement seems to have led to notable under-reporting on the important fruit tree *Borassus aethiopum* Mart—the tall savanna palm posing a daunting prospect for the most determined botanical collector.

In preparing this study for publication twenty years after the data collection, the author worked from the original entries and notes made by the interviewers, ranging from sparse initial entries to more expansive and anecdotal accounts. In many cases this involved interpretation, particularly as regards the body systems involved in a particular ailment, such as ‘stomach problems’ or ‘eye problems’ which have been left as such in Table 2, although according to the Relative Importance Index metric described above, the author was obliged to provide interpretations as to the number of pharmacological properties attributed to each taxon, and the number of body systems within which these properties were active. Because these interpretations could no longer be checked with the respondents, this may represent a source of error in the rankings.

An example of (apparently) purely ritual function, bumping the head of a mumps sufferer on a bough of *Kigelia africana*, was included in Table 2 as a medical treatment *prima facie*—whereas another example, exorcism of spirits from a patient by in taking by inhalation of leaf volatiles in steam, was subject to author interpretation as a treatment for psychosis. Otherwise, maladies and conditions of indication are left as recorded, representative as they are of the emic (respondent cultural) perspective.

## Conclusions

The study provides an inventory of medicinal plant use applications and an account of the indigenous ethnopharmacological knowledge held within four cultures of northern Uganda, at an historical moment before conflict and dislocation disrupted the transmission of traditional knowledge to the current generation. During the two-decade interval since the data were collected, Uganda has produced its share or better of ethnobotanical studies, reflecting a relatively high level of awareness and an enabling policy environment, which provide a degree of ethnobotanical, pharmacological and phytochemical corroboration of the majority of reported use applications, including some around the periphery of the study area. Of the 309 sources consulted here, 224 (72%) were published during the last 20 years.

The ethnopharmacological use of plants in the Acholi, Teso and Ethur cultures of Uganda have previously not been documented in earlier ethnopharmacological studies—to date, a lacuna comprising the Nilotic cultural family, within northern Uganda and contiguous areas of South Sudan—so their inclusion here contributes new data to the field in that respect. By comparison to the earlier Uganda studies, this paper presents a more comprehensive review of several types of corroborating literature, distinguishing ethnobotanical uses from pharmacological and compositional data, drawing deeply from an extensive body of literature including ethnobotanical, pharmacological and phytochemical studies. As such, this paper will assist in extending interpretation of the historical Ugandan *corpus*.

While the results of the study are largely consistent with earlier ethnobotanical studies of the Africa region as a whole, and a majority of reported use applications reflected in the literature, a significant proportion of the use reports (on the order of 27%) cannot be corroborated by those earlier studies, though pharmacological and phytochemical data for some taxa show promise. These taxa, in particular, should be investigated further by future researchers. Future studies should likewise seek to determine whether the body of knowledge presented here is still held within the study area, and whether use patterns and frequency of use described by the respondents have changed over time.

It has been suggested that the environmental knowledge of rural communities can provide a foundation upon which to establish collaborative conservation and management of plant biodiversity [8], and that cultural comparisons of ethnobiological knowledge provide a basis for conservation of species through locally-driven resource management [151]. As such, it is hoped that the results of this study may contribute to these long-term outcomes.

### Supplementary Information

**Additional file 1.** Cross-referencing of the data provided in Table 2 with the findings of earlier ethnobotanical studies on medicinal applications, and with pharmacological and compositional studies on bioactivity and compounds of interest.

## Data Availability

The dataset supporting the conclusions of this article is included within the article and its additional file.

## Abbreviations

ARIs: Acute Respiratory Infections
COX-1: Cyclooxygenase-1
GABA: γ-Aminobutyric acid
HIV: Human Immunodeficiency Virus
JI: Jaccard Index of Similarity
MHU: Makerere University Herbarium
PL: Phospholipase
PROSEA: Plant Resources of South-East Asia
RSI: Rahman’s Similarity Index
RNA: Ribonucleic acid
RI: Relative Importance Index
RSV: Respiratory Syncytial Virus
STD: Sexually-Transmitted Disease
UR: Use Report
WHO: World Health Organization

## Footnotes

1 Also spelled as Aswaa, Acwa, Acwaa.
